# Multi-Omics Association Reveals the Effects of Intestinal Microbiome–Host Interactions on Fat Deposition in Broilers

**DOI:** 10.3389/fmicb.2021.815538

**Published:** 2022-02-17

**Authors:** Yang Jing, Yuqi Yuan, Melissa Monson, Peng Wang, Fang Mu, Qi Zhang, Wei Na, Ke Zhang, Yuxiang Wang, Li Leng, Yumao Li, Peng Luan, Ning Wang, Rongjun Guo, Susan J. Lamont, Hui Li, Hui Yuan

**Affiliations:** ^1^Key Laboratory of Chicken Genetics and Breeding, Ministry of Agriculture and Rural Affairs, Harbin, China; ^2^Key Laboratory of Animal Genetics, Breeding and Reproduction, Education Department of Heilongjiang Province, Harbin, China; ^3^College of Animal Science and Technology, Northeast Agricultural University, Harbin, China; ^4^Novogene Bioinformatics Institute, Beijing, China; ^5^Department of Animal Science, Iowa State University, Ames, IA, United States

**Keywords:** whole metagenome sequencing, mRNA sequencing, multi-omics associations, obesity, broiler

## Abstract

Growing evidence indicates that gut microbiota factors cannot be viewed as independent in the occurrence of obesity. Because the gut microbiome is highly dimensional and complex, studies on interactions between gut microbiome and host in obesity are still rare. To explore the relationship of gut microbiome–host interactions with obesity, we performed multi-omics associations of gut metagenome, intestinal transcriptome, and host obesity phenotypes in divergently selected obese–lean broiler lines. Metagenomic shotgun sequencing generated a total of 450 gigabases of clean data from 80 intestinal segment contents of 20 broilers (10 of each line). The microbiome comparison showed that microbial diversity and composition in the duodenum, jejunum, ileum, and ceca were altered variously between the lean- and fat-line broilers. We identified two jejunal microbes (*Escherichia coli* and *Candidatus Acetothermia bacterium*) and four cecal microbes (*Alistipes* sp. *CHKCI003*, *Ruminococcaceae bacterium CPB6*, *Clostridiales bacterium*, and *Anaeromassilibacillus* sp. *An200*), which were significantly different between the two lines (FDR < 0.05). When comparing functional metagenome, the fat-line broilers had an intensive microbial metabolism in the duodenum and jejunum but degenerative microbial activities in the ileum and ceca. mRNA-sequencing identified a total of 1,667 differentially expressed genes (DEG) in the four intestinal compartments between the two lines (| log2FC| > 1.5 and FDR < 0.05). Multi-omics associations showed that the 14 microbial species with abundances that were significantly related with abdominal fat relevant traits (AFRT) also have significant correlations with 155 AFRT-correlated DEG (*p* < 0.05). These DEG were mainly involved in lipid metabolism, immune system, transport and catabolism, and cell growth-related pathways. The present study constructed a gut microbial gene catalog of the obese–lean broiler lines. Intestinal transcriptome and metagenome comparison between the two lines identified candidate DEG and differential microbes for obesity, respectively. Multi-omics associations suggest that abdominal fat deposition may be influenced by the interactions of specific gut microbiota abundance and the expression of host genes in the intestinal compartments in which the microbes reside. Our study explored the interactions between gut microbiome and host intestinal gene expression in lean and obese broilers, which may expand knowledge on the relationships between obesity and gut microbiome.

## Introduction

Obesity is a systemic lipodystrophic syndrome which is a serious health problem worldwide in humans. Chicken has unique metabolic features, i.e., hyperglycemia and insulin resistance, which can serve as an interesting model organism for studying the development of obesity (Resnyk et al., 2013). In chicken, excessive abdominal fat accumulation is the major obesity phenotype, which causes negative influence on chicken production and consumption (Wang et al., 2007; Abdalla et al., 2018). Growing evidence has proved that crucial gut microbiome alteration could lead to many chronic metabolic disorders (Yan et al., 2017; Aw and Fukuda, 2018; Hoyles et al., 2018), especially in adiposity (Bouter et al., 2017). Although previous studies have indicated that gut microbiota had comprehensive impacts on obesity (Ussar et al., 2016; Org and Lusis, 2018; Lee et al., 2020), the underlying interaction mechanism between gut microbiome and host remains ambiguous.

The animal intestinal tract, as an important interface between host and gut microenvironment, functions as an organ for digestion and absorption of diets (Parker and Picut, 2016). The intestinal tract functional variations directly impact systemic nutrition metabolism, especially lipid metabolism, which ultimately contributes to obesity (Kondo et al., 2006; Mao et al., 2013). Altered functions such as an increase in mucus layer permeability and breach of intestinal integrity have been reported in obese mice, though causality was unclear (Teixeira et al., 2012; Araujo et al., 2017). At the molecular level, several intestinal genes and pathways were also significantly changed in obese humans and mice, compared to controls (Mao et al., 2013; Pfalzer et al., 2016; Xie et al., 2017). Altered intestinal functions could be mediated by the varied gut microbiome in obesity. Previously, studies of human fecal microbiome have found that obese individuals possessed significantly decreased microbial diversity and metagenome gene counts (Turnbaugh et al., 2009; Le Chatelier et al., 2013). When comparing gut microbial composition between obese and lean individuals, the differences of specific microbial relative abundance were inconsistent among studies, with some researchers reporting a higher ratio of Firmicutes to Bacteroidetes (F/B) in obesity (Turnbaugh et al., 2006, 2009; Koliada et al., 2017), whereas other studies have not drawn a similar conclusion without any explanation (Duncan et al., 2008; Schwiertz et al., 2010b; Fernandes et al., 2014). In regard to microbial relative abundance, genera including *Megamonas* (Maya-Lucas et al., 2019), *Oscillospira*, *Ruminococcus* (Jiao et al., 2018), *Fusobacterium*, *Escherichia-Shigella*, and *Pseudomonas* (Gao et al., 2018) were significantly increased in obese humans and mice. Recently, studies on chicken cecal microbiome showed that *Microbacterium*, *Sphingonomas*, *Olsenella*, *Methanobrevibacter*, and *Slackia* were positively correlated with fat metabolism (Xiang et al., 2021; Zhang et al., 2021). Functional analysis of microbiome indicated that pyruvate metabolism, butanoate metabolism, propanoate metabolism, pentose phosphate pathway, fatty acid biosynthesis, and glycerolipid metabolism pathways were enriched in obese mice (Jiao et al., 2018), while glycolysis I (from glucose 6-phosphate) and glycolysis II (from fructose 6-phosphate) were significantly overrepresented in normal individuals (Maya-Lucas et al., 2019). In chicken, functional enrichments of cecal microbiome found that microbial pathways including lipid metabolism, carbohydrate metabolism, and energy metabolism pathways were upregulated in high AFD group, when compared to low-AFD group (Xiang et al., 2021).

In general, most studies were only focused on gut microbiome differences between obesity and control and ignored the interactions between the gut microbiota and host. However, a previous study in chickens implied that gut microbiome could not be viewed as independent of its environment and is regulated by the host genetic background (Zhao et al., 2013). Considering the proximity and direct contact between the intestine and the microbiota, we hypothesized that the interaction of the specialized intestinal microbiome, which resulted from genetic selection, with the intestinal compartment in which it resides is an important mechanism contributing to AFD in broilers. In this study, multi-omics combination analysis of host phenotype, intestinal transcriptome, and corresponding metagenome in the Northeast Agricultural University broiler lines divergently selected for abdominal fat content (NEAUHLF) has been performed to reveal the association of host–gut microorganism interactions with AFD. These findings may benefit our understanding of gut microbial effects on obesity and offer novel guidance for strategies to target gut–microbe interactions to mitigate abdominal fat accumulation in broilers.

## Materials and Methods

### Experimental Population

The lean and fat broilers were derived from the 21st generation of the NEAUHLF. We have previously reported the detailed breeding scheme of NEAUHLF (Guo et al., 2011). After 21 generations of selection, there is over a 10-fold difference in AFP between the two lines, representing a classical obese–lean study model. All broilers were raised in similar feeding surroundings and had free access to feed and water. To prevent contamination from uncontrolled particle intake and feathers, all broilers were raised in individual cages with wire floors. A commercial soybean-based diet that met all of the National Research Council (NRC) requirements was provided; a starter diet of 3,000 kcal ME/kg and 210 g/kg CP was fed to the birds until 3 weeks of age, while a grower diet of 3,100 kcal metabolizable energy (ME)/kg and 190 g/kg crude protein (CP) was fed from 3 to 7 weeks of age (Guo et al., 2011). No antibodies nor veterinary drugs were used in animal raising of the present study. At age of 7 weeks, 10 male individuals of each line were selected randomly to investigate the interactions between gut microbiome, host gene expression, and obesity.

### Phenotypic Measurement and Sample Collection

After fasting 12 h, the BW of the 20 experimental birds were measured with an electronic scale. Blood was collected from the brachial wing vein, and serum fraction was sampled for measurement of serum biochemical parameters using an Architect C8000 Automatic Biochemical Analyzer (Abbott, Inc., Chicago, IL, United States) in the clinical laboratory of the Fourth Hospital of Harbin Medical University (Harbin, China). The measurements included TBA, TP, TG, GLU, CHO, HDL-C, LDL-C, GGT, AST, ALT, ALB, UA, and CREA (Dong et al., 2015). Then the birds were euthanized, and the AFW was measured, while the AFP was calculated based on the AFW and BW. Four intestinal sections including the duodenum, jejunum, ileum, and ceca were carefully divided with sterilized monofilament nylon thread, placed on ice, and rapidly transported to the laboratory for further processing. The duodenal compartment starts at the pylorus of the gizzard and extends to the end of the duodenal loop; the jejunal compartment follows the duodenum and extends to the Meckel’s diverticulum, while the ileal compartment follows the jejunum and extends to the ileocecal junction, followed with cecal sections in the two flanks (Wang et al., 2014). The four intestinal contents were sampled for metagenome sequencing following the procedure reported by Quince et al. (2017). To avoid contamination bias, the luminal contents were sampled and frozen promptly with liquid nitrogen in the laboratory super clean bench. Subsequently, 3 cm of intestinal tissue from the middle of each section was sampled and snap-frozen in liquid nitrogen for mRNA sequencing.

### Metagenome DNA Preparation and Sequencing

The gut microbial DNA was isolated from the four intestinal contents per bird using the QIAamp DNA Stool Mini Kit (Qiagen, Hilden, Germany) following the standard manufacturer’s protocol at Novogene Bioinformatics Technology Co., Ltd (Tianjin, China) (Lim et al., 2018). In DNA quality control, Optical Density (OD) value between 1.8 and 2.0 and DNA yields above 1 μg were prepared for each library construction. The qualified DNA samples were randomly interrupted into about 350-bp fragments using a Covaris sonicator (Covaris, Inc., Woburn, MA, United States). Then, to prepare these fragments for Illumina sequencing using PCR amplification, samples were end-polished, polyA-tailed, and ligated with the full-length adaptors (Illumina Inc., San Diego, CA, United States). The AMPure XP system (Beckman Coulter, Beverly, MA, United States) was utilized to purify the PCR products, while the Agilent 2100 Bioanalyzer (Agilent Technologies, Santa Clara, CA, United States) and real-time PCR were applied to evaluate size distribution and quantity of libraries at Novogene Bioinformatics Technology Co., Ltd, respectively. After clustering of the index-coded samples on the flow cells, the libraries were sequenced on the Illumina PE150 platform (Novogene Bioinformatics Technology Co., Ltd.) and 150-bp paired-end reads were generated.

### Metagenome Assembly and Gene Catalog Construction

Readfq software (V8^1^) was performed to preprocess the raw data, from which unqualified reads and adapters were filtered out to obtain clean data. Considering the possibility of host and food contamination (Huang et al., 2018), clean data were subsequently aligned to the possible non-microbial genomes (including chicken, human, corn, wheat, and soybean genome) using Bowtie2.2.4 software to filter out the noisy reads (from contaminating genomes) with the default parameters (Langmead and Salzberg, 2012). For single-sample assembly, the clean reads from each sample were then assembled and analyzed by SOAPdenovo (V2.04, parameters: -d 1, -M 3, -R, -u, -F, -K 55) (Luo et al., 2012; Qin et al., 2014; Feng et al., 2015). The assembled scaffolds were separated at connecting Ns, leaving scaftigs without Ns (Mende et al., 2012; Qin et al., 2014). To maximize the utilization of data, a mixed sample assembly process was performed on the unused PE reads that did not align to the scaftigs using Bowtie2.2.4 (Qin et al., 2014); the unused reads from all samples were combined and used to assemble additional scaftigs using SOAPdenovo. Open reading frame (ORF) prediction was performed for all scaftigs longer than 500 bp using MetaGeneMark software (V2.10^2^); any predicted ORFs shorter than 100 nt were removed with default parameters (Qin et al., 2010; Li et al., 2014). The unique initial gene catalog was constructed through CD-HIT software (V4.5.8; parameters: -c 0.95, -G 0, -aS 0.9, -g 1, -d 0) (Fu et al., 2012). The clean data of each sample were then mapped to the initial catalog using Bowtie2.2.4 with default parameters to obtain the number of reads per gene in each sample (Li et al., 2014; Qin et al., 2014). Genes with less than two aligned reads in each sample were removed to acquire the final gene catalog, which was applied in subsequent analysis (Li et al., 2014). According to the gene length and the mapped read counts, the abundance of each gene (*G* in the formula below) in each sample was statistically calculated by the following formula:


$${\text{G}}_{k}-\frac{r_{k}}{L_{k}}\times \frac{1}{\sum_{i=1}^{n}\frac{r_{i}}{L_{i}}}$$

in which *r* stands for the mapped read counts of each gene and *L* is the corresponding gene length (Qin et al., 2010). The above analyses were performed by Novogene Bioinformatics Technology Co., Ltd.

### Taxonomy Prediction and Statistics

DIAMOND software (V0.9.9^3^) was adopted to BLAST the catalog genes to the NR database (Version: 2019-04-09^4^) of National Center for Biotechnology Information (NCBI) with the parameter of blastp, -e 1e-5. As multiple aligned results for one Unigene, the lowest common ancestor (LCA) algorithm of MEGAN software was applied to acquire the final taxon annotation of each Unigene (Oh et al., 2014). The final chicken gut MGC included 1,296,491 genes. On the basis of gene abundance and LCA annotations, the quantity and abundance matrix of each taxonomy hierarchy (kingdom, phylum, class, order, family, genus, and species) were calculated statistically per sample. The above analyses were performed by Novogene Bioinformatics Technology Co., Ltd. Based on the above taxonomy hierarchy matrix, the subsequent analysis of microbiota structure and diversity was performed with the vegan package (Oksanen et al., 2013) in R (version 3.6.1). The Metastats method compiled with R language was performed in detection of differential microbial taxa between two lines. A permutation test between the two lines was used in Metastats analysis for each taxonomy, while Benjamini and Hochberg false discovery rate (FDR) was used to correct each *p*-value (White et al., 2009). Taxonomies identified in all samples of at least one group and with relative abundance over 0.1% were used to perform the differential comparisons between two lines.

### Metagenome Functional Annotation

DIAMOND software (V0.9.9) was also performed to BLAST the catalog genes to the Kyoto Encyclopedia of Genes and Genomes database (KEGG, Version 20180101), Carbohydrate-Active enZYmes database (CAZY, Version 2015.08) and evolutionary genealogy of genes: Non-supervised Orthologous Groups database (eggNOG, Version 4.5) with the parameter setting of blastp, -e 1e-5 (Feng et al., 2015). For multiple BLAST results of each Unigene, the best BLAST hit was selected as the final function annotation (Feng et al., 2015). The above analyses were carried out by Novogene Bioinformatics Technology Co., Ltd. For the functional abundance matrix, the relative abundance of each functional hierarchy was the sum of the relative abundances for genes annotated to that functional level per sample.

### Transcriptome Sequencing and Analysis

The total RNA of the corresponding four intestinal tissues was prepared for mRNA sequencing using TRizol reagent (Invitrogen, Carlsbad, CA, United States) following the manufacturer’s protocol at Novogene Bioinformatics Technology Co., Ltd. The RNA integrity and yield were assessed by the RNA Nano 6000 Assay Kit of the Bioanalyzer 2100 system (Agilent Technologies, Santa Clara, CA, United States) and the NanoPhotometer^®^ spectrophotometer (IMPLEN, Westlake Village, CA, United States), respectively. The RNA Integrity Number values were all greater than 6.8 (average of 8.28), and the RNA yield of each sample was more than 4 μg. In accordance with the manufacturer’s protocol of the NEBNext^®^ Ultra™ RNA Library Prep Kit for Illumina^®^ (NEB, Ipswich, MA, United States), 3 μg of RNA per sample was used to generate sequencing libraries with index codes added to assign sequences to each sample. The libraries were clustered following the manufacturer’s recommendations of the cBot Cluster Generation System using TruSeq PE Cluster Kit v3-cBot-HS (Illumina Inc.). Finally, the clustered libraries were sequenced on an Illumina HiSeq platform (by Novogene Bioinformatics Technology Co., Ltd.), and 150-bp paired-end reads were generated.

Reads containing adapter, reads containing poly-N, and low-quality reads from raw data were filtered using Trimmomatic with the default parameters (PE ILLUMINACLIP:./adapter_fasta.fa:2:30:10 SLIDINGWINDOW:4:15 LEADING:3 TRAILING:3 MINLEN:36) (Bolger et al., 2014). Subsequently, high quality clean data were mapped to the chicken reference genome (version: galGal5). Reference genome and gene model annotation files were downloaded from the NCBI genome website directly. Bowtie v2.2.3 was employed to build a search index of the reference genome, then TopHat v2.0.12 was applied to align the pair-end clean reads to the reference genome (Trapnell et al., 2009). The reads mapped to each gene in each individual dataset were counted by using HTSeq v0.6.1 (Anders et al., 2015). The above steps of read mapping and counts were performed by the Novogene Bioinformatics Technology Co., Ltd. Differential expression analysis of the lean vs. fat lines within each intestinal compartment was performed using the DESeq2 R package (version 1.24.0) (Love et al., 2014). Genes with FDR (adjusted *p*-value) less than 0.05 and absolute log2-fold change more than 1.5 were assigned as DEGs. For gene functional enrichment analysis, KEGG enrichment was subsequently performed using the clusterProfiler R package (v3.12.0) and the pathway profiles in the KEGG database (Yu et al., 2012).

### Differentially Expressed Genes Validation Using qPCR

The RNA isolates used for qPCR validation were the same as those used for RNA-seq. The PrimeScript™ RT reagent kit with gDNA Eraser (Perfect Real Time) (Takara Bio Inc., Kusatsu, Japan) was applied to remove genomic DNA and perform the reverse transcription reaction following the manufacturer’s recommended protocol. Specific primers (see in Supplementary Table 1) for candidate genes with the ideal amplification efficiency between 90% and 110% were selected to perform the validated experiment (Broeders et al., 2014). The ABI QuantStudio™ 6 Flex Real-Time PCR System (Applied Biosystems, Foster City, CA, United States) was adopted to perform the real-time qPCR of candidate genes with FastStart SYBR Green Master (Roche, Mannheim, BW, Germany). The reaction conditions were as follows: an initial denaturation at 95°C for 10 min, 40 cycles of 15 s at 95°C, and 1 min at 60°C. Internal reference gene stability was estimated with NormFinder R scripts^5^, and *TBP* was finally selected as the internal reference gene. Ct values of all genes were analyzed using QuantStudio™ Real-Time PCR Software v1.3 (Applied Biosystems). Gene expression comparison between the two lines within each intestinal compartment was done using the 2^–ΔΔ*Ct*^ method (Livak and Schmittgen, 2001) in Excel and pairwise correlation and linear regression between Log2FC (–ΔΔCt for each comparison) in qPCR validation, and Log2FC in mRNA-Seq were calculated in JMP 10.0.0 (SAS Institute, Inc., Cary, NC, United States).

### Statistical Analysis and Multi-Omics Association

Phenotypes were statistically compared to the difference between the lean and fat broilers with Student’s *t*-test in SPSS 18.0 (IBM, Armonk, NY, United States). Omics-related analysis were performed in R software, version 3.6.1. Phenotype correlations were firstly calculated, and serum biochemical indices (ALB, CHO, GGT, GLU, HDL-C, HDL-C/LDL-C, TP, UA) that were significantly related with AFW or AFP were considered AFRTs (Supplementary Figure 1). To obtain the AFRT-correlated genes and microbial species within each tissue compartment, we first employed correlations of the intestinal transcriptome and gut microbiome with AFRT using the weighted gene co-expression network analysis (WGCNA) R package (Miller et al., 2010). In detail, genes of the transcriptome were first clustered into multi-modules, and modules that significantly correlated (Pearson correlations) with AFRT were selected (Supplementary Figure 2). Subsequently, genes in the above modules were screened for individual correlations with AFRT (*p*-values less than 0.05 identified AFRT-correlated genes). Finally, hub genes that correlated with AFRT were selected to perform the following multi-omics correlations (Supplementary Table 2). As there was non-normal distribution of microbial data, Spearman rank correlations were adopted when calculating correlations with microbial species. Otherwise, similar analysis procedures in WGNCA were performed in selecting AFRT-correlated microbial species (Supplementary Figure 3). Spearman rank correlations were finally performed to identify the correlations between AFRT-correlated microbial species and host genes in each tissue; genes with less *p*-values than 0.05 were viewed as significantly correlated with AFRT-correlated species. Because there was a far greater number of species in the cecal microbiome than in the small intestine, only microbes in the ceca that significantly differed between the two lines were correlated with the AFRT, and a more stringent threshold of *p*-value less than 0.01 was used for the final correlation of AFRT-correlated genes and microbe.

## Results

### Phenotype Characterization of the Lean- and Fat-Line Broilers

The comparison analysis of the phenotypes between two lines are summarized in Table 1. The studied broilers from two lines had a significant difference of AFW and AFP (*p* < 0.05), with no difference of BW (*p* > 0.05). For clinical serum biochemical phenotypes, HDL-C, ratio of HDL-C to LDL-C, TP, ALB, GGT, and UA in the fat line were significantly higher than in the lean line, while GLU was significant decreased in fat-line broilers (*p* < 0.05). These phenotypes indicated that this sample of broilers satisfactorily represents our NEAUHLF population as an ideal obese–lean study model to investigate our hypothesis.

**TABLE 1:** The comparison of phenotypes between the lean and fat lines.

Phenotype	Lean line (*n* = 10)	Fat line (*n* = 10)	*p*-Value
BW/kg	2.218 ± 0.015	2.189 ± 0.129	0.370
AFW/g	14.319 ± 1.127^b^	117.869 ± 4.306^a^	1.037 × 10^–13^
AFP/%	0.646 ± 0.050^b^	5.401 ± 0.210^a^	3.507 × 10^–13^
TG (mmol/L)	0.322 ± 0.023	0.378 ± 0.029	0.835
CHO (mmol/L)	3.051 ± 0.091	3.271 ± 0.111	0.425
HDL-C (mmol/L)	2.164 ± 0.071^b^	2.573 ± 0.094^a^	0.002
LDL-C (mmol/L)	0.649 ± 0.036	0.591 ± 0.039	0.282
HDL-C/LDL-C (%)	3.173 ± 0.303^b^	4.658 ± 0.432^a^	0.017
TBA (μmol/L)	2.743 ± 0.484	2.943 ± 0.610	0.799
TP (g/L)	29.607 ± 0.653^b^	36.657 ± 1.369^a^	8.5 × 10^–5^
ALB (g/L)	13.143 ± 0.310^b^	15.157 ± 0.494^a^	0.002
GLU (mmol/L)	12.174 ± 0.132^a^	10.761 ± 0.225^b^	1.1 × 10^–5^
AST (U/L)	288.786 ± 12.894	265.000 ± 13.903	0.221
ALT (U/L)	2.500 ± 0.272	2.286 ± 0.194	0.527
AST/ALT (%)	143.232 ± 25.858	132.357 ± 16.592	0.726
CREA (μmol/L)	3.157 ± 0.329	3.750 ± 0.379	0.248
GGT (U/L)	14.357 ± 0.862^b^	19.071 ± 0.863^a^	0.001
UA (μmol/L)	162.743 ± 17.384^b^	284.350 ± 39.886^a^	0.010

*^a,b^Superscript letters in the same row mean a significant difference between the two lines (p < 0.05). Values in table are described with mean ± standard error. BW, body weight; AFW, abdominal fat weight; AFP, abdominal fat percentage; TG, triglycerides; CHO, total cholesterol; HDL-C, high-density lipoprotein cholesterol; LDL-C, low-density lipoprotein cholesterol; TBA, total bile acid; TP, total protein; ALB, albumin; GLU, glucose; AST, aspartate transaminase; ALT, alanine transaminase; CREA, creatinine; GGT, γ-glutamyl transpeptidase; UA, uric acid.*

### Gut Microbial Gene Catalog

To profile the gut metagenome, we sampled 80 intestinal contents from the four intestinal compartments (duodenum, jejunum, ileum, and ceca) of the same 10 broilers per line. Metagenomic shotgun sequencing was performed to generate a total of 450 gigabases (Gb) of clean data (average of 5.63 Gb per sample) (Supplementary Table 3). A total of 2.44 million non-redundant genes were then identified, with an average ORF length of 560.44 bp (details in Supplementary Table 4). In rarefaction analysis on all samples, identified microbial genes (MG) numbers approached saturation by 20 samples and, when each tissue was considered separately, revealed that the majority of MG were identified in the ceca. These results indicated that the gut MGC has covered most of the microbiota in our broilers (Supplementary Figure 4).

Microbial taxa annotation showed that over half of the non-redundant genes (1.29 million) could be taxonomically classified (details in Supplementary Table 5). In total, 99.7% genes with annotations were taxonomically assigned to the kingdom level, among which bacteria account for 99.4% of the annotated genes, with 0.3% being from archaea and eukaryote and 0.3% being from virus and unclassified kingdom. Of bacterial genes, over 93% genes belonged to the main four phyla, including Firmicutes (79.5%), Bacteroidetes (9.0%), Proteobacteria (4.1%), and Actinobacteria (0.5%). At lower taxonomic levels, 71.91% and 57.18% of the annotated genes in this catalog were taxonomically classified at the genus and species levels, respectively. At the genus levels, most of the bacterial genes annotated to *Clostridium* (15.76%), followed by *Lachnoclostridium* (7.03%), *Bacteroides* (5.56%), *Flavonifractor* (4.52%), and *Blautia* (3.73%). At the species levels, most bacterial genes belonged to *Firmicutes bacterium* (7.57%), followed by *Clostridiales bacterium* (4.42%), *Lachnoclostridium* sp. *An298* (1.30%), *Candidatus Borkfalki ceftriaxensis* (1.19%), and *Pseudoflavonifractor* sp. *An184* (1.11%).

### The Gut Microbiome Composition Analysis of the Lean- and Fat-Line Broilers

**FIGURE 1:**
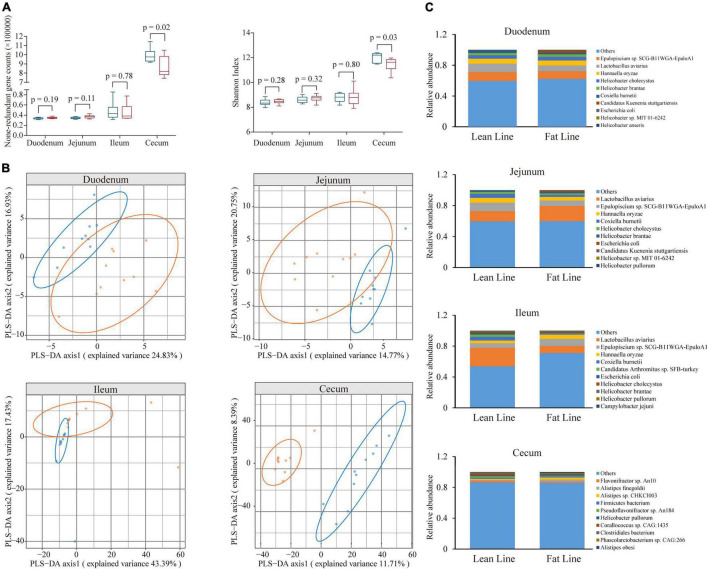
The difference of microbial diversity and composition between the lean- and fat-line broilers. **(A)** The comparison of non-redundant gene numbers and Shannon index in four intestinal compartments between the two lines. Mann–Whitney *U* test was performed to verify the difference, in which *p* < 0.05 indicated a significant difference. **(B)** Partial least squares discriminant analysis of microbial species composition in the four intestinal compartments, which showed a clear isolation of the ceca and some extent of separation in the small intestine (duodenum, jejunum, and ileum) between the two lines. **(C)** The relative abundance of the top 10 microbial species in the four intestinal compartments of the two lines of broilers. The blue boxes and points represent lean-line broilers, while the red boxes and points are fat-line broilers.

**FIGURE 2:**
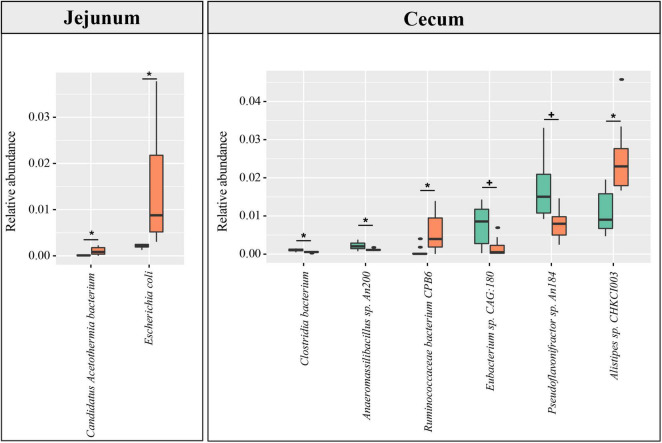
The difference in species abundance between the lean- and fat-line broilers. Microbial species identified in all samples of at least one group with relative abundance over 0.1% were used to perform the differential comparison between the two lines. Metastats software was used, and species with FDR < 0.1 are shown in the figure. No differential species between the two broiler lines were identified in the duodenum and ileum. The green and orange columns stand for lean line and fat line, respectively. The green boxes stand for lean-line broilers, while the orange boxes stand for fat-line broilers. *FDR < 0.05; ^+^0.05 ≤ FDR < 0.1.

Based on the constructed MGC, the relative abundance matrix of microbial taxa was calculated to investigate the gut microbiota differences between the lean and fat broilers. In microbial alpha diversity, the fat-line broilers revealed a significant decrease in both gene counts and Shannon index in the ceca compared with the lean-line broilers (*p* < 0.05), and there were no significant differences between lines in the small intestine (*p* > 0.05, Figure 1A). To avoid confounding variations within group, partial least squares discriminant analysis (PLS-DA) was performed as a supervised model to illustrate the microbial species variations between the two lines for each tissue compartment, which showed that there was some extent of separation between the two lines, although with partial overlap, in small intestinal compartments and a clear separation of each line in the ceca (Figure 1B). The relative abundance of dominant species in the four intestinal compartments of each line is displayed in Figure 1C. Subsequently, microbes identified in all samples of at least one group with relative abundance exceeding 0.1% were compared in the four intestinal compartments between the two lines; this showed that the jejunum and ceca had a distinct microbial characterization at both genus and species levels (Figure 2 and Supplementary Figure 5). At the species level, *Escherichia coli* and *Candidatus Acetothermia bacterium* were significantly increased in the jejunum of fat-line broilers (FDR < 0.05, Figure 2). In the ceca, two microbial species, *Alistipes* sp. *CHKCI003* and *Ruminococcaceae bacterium CPB6*, had a significantly increased abundance in fat-line broilers, whereas two other species (*C. bacterium* and *Anaeromassilibacillus* sp. *An200*) were significantly decreased (FDR < 0.05, Figure 2). Another two cecal species, *Pseudoflavonifractor* sp. *An184* and *Eubacterium* sp. *CAG:180*, showed an increased trend in the lean line (FDR < 0.1, Figure 2). As the F/B is a candidate indicator for obesity, we also examined the F/B, which was decreased in all four intestinal compartments of obese birds (Supplementary Figure 6).

### Functional Metagenome Profile of the Lean- and Fat-Line Broilers

**FIGURE 3:**
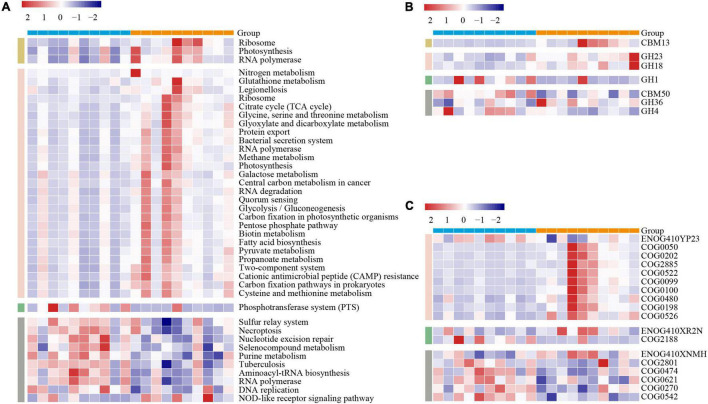
Variations of the gut microbiome functions between the lean- and fat-line broilers. The functional categories of KEGG pathways **(A)**, carbohydrate-active enZYmes families **(B)**, and eggNOG ortholog groups **(C)** were compared between the lean and fat lines through non-parametric Mann–Whitney *U* test, in which *p*-values less than 0.05 were shown in the figure. These functional categories that participated in the comparison existed in all samples of at least one line with relative abundances that were over 0.1%. The color scale represents the row *Z* score. The blue and orange patterns in the upper bound of each heatmap are the individuals of lean line and fat line, respectively. The yellow, pink, green, and gray patterns in the left part of each heatmap represent the duodenum, jejunum, ileum, and ceca, respectively.

To further gain the functional metagenome variations between the two lines of broilers, we subsequently annotated the MG by using three gene functional databases: KEGG, CAZY, and eggNOG. To avoid biases caused by varied sequencing depth among samples, we screened the functional categories that existed at least in all individuals of one line with the mean relative abundance over 0.1%. In KEGG pathways comparison, we, respectively, identified 3, 27, 1, and 10 pathways of duodenum, jejunum, ileum, and ceca significantly varied between the two lines of broilers (*p* < 0.05, Figure 3A). The jejunum occupied more numbers of significantly differential microbial pathways between the two lines, including fatty acid metabolism, varied amino acids metabolism, carbohydrate metabolism, membrane transport, nucleic acids metabolism, biotin metabolism, and energy metabolism (*p* < 0.05, Figure 3A). In the ceca, nine microbial pathways mainly containing nucleotide metabolism and amino acid metabolism were significantly increased in the lean line, while “NOD-like receptor signaling pathway” was significantly enriched in the fat line (*p* < 0.05, Figure 3A).

The CAZY families comparison showed that seven families in the four intestinal compartments were significantly different between the two lines (duodenum, 1; jejunum, 2; ileum, 1; ceca, 3; *p* < 0.05, Figure 3B). CBM13, as a kind of carbohydrate-binding module-binding mannose, was significantly increased in the duodenum of the fat-line broilers (*p* < 0.05, Figure 3B). The glycoside hydrolases (GH23 and GH18) were significantly enhanced in the jejunum of the fat-line broilers (*p* < 0.05, Figure 3B). The two glycoside hydrolase families were involved in cleaving either chitin or peptidoglycan, which demonstrated that the jejunal microbiome of fat-line broilers displayed increased activities of turnover. GH1, as a family of glycoside hydrolases containing glucosidase, galactosidase, mannosidase, etc., was found to significantly increase in the ileum of the lean-line broilers (*p* < 0.05, Figure 3B). Among the three CAZY families in the ceca, CBM50 and GH4 were significantly enriched in the lean-line broilers, while GH36 was significantly enhanced in the fat-line broilers (*p* < 0.05, Figure 3B).

A total of 18 significantly differential ortholog groups were identified in all intestinal compartments except for the duodenum when compared in eggNOG terms (jejunum, 10; ileum, 2; ceca, 6; *p* < 0.05, Figure 3C). In the jejunum, apart from ENOG410YP23 (related with ubiquinone metabolism), the other nine ortholog groups (mainly involved in protein biosynthesis and modification) were significantly enriched in the fat-line broilers (*p* < 0.05, Figure 3C). For the ileum, ENOG410XR2N [lysine (K)-specific demethylase] was significantly enriched in the fat line, while COG2188 (GntR family transcriptional regulator) was significantly enhanced in the lean line (*p* < 0.05, Figure 3C). Among the six ortholog groups in the ceca, only ENOG410XNMH (histidine kinase) was significantly enriched in the fat line; the other five microbial ortholog groups (COG2801, COG0474, COG0621, COG0270, and COG0542) were significantly enriched in the lean line (*p* < 0.05, Figure 3C). These five ortholog groups are mainly associated with retrotransposon, energy metabolism, translation, and methylation. These functional comparisons of gut microbiome demonstrated that fat birds have enhanced microbial activities (e.g., carbohydrate and protein metabolism) in the duodenum and jejunum but are hypoactive in the ileum and cecum.

### The Intestinal Transcriptome Landscapes of the Lean- and Fat-Line Broilers

The mRNA sequencing of 80 samples from four intestinal compartments were profiled through Illumina HiSeq platform. A total of 4,210,120,566 raw reads with a length of 150 bp were obtained from the sequencing of 80 libraries. After quality control, a total of 4,034,423,542 clean reads were retained with the average clean data yield of 7.56 GB per sample, and the percentage of Q30 bases was > 90% (Supplementary Table 6). The rates of clean reads mapped to the chicken reference genome (version: galGal5) have been summarized in Supplementary Table 6, of which the average mapping rate was more than 80%. Meanwhile, approximately 20,000 chicken genes were detected in each sample (Supplementary Table 6), and in total, 28,199 genes were detected. Among these genes, a total of 26,309 genes were identified as known genes, with the remaining 1,890 novel genes.

Based on the read counts matrix, the DESeq2 R package was applied to identify the DEG of the four intestinal compartments between the lean and fat lines. As shown in Supplementary Figure 7, more downregulated DEG were identified in the lean broilers from all four intestinal compartments using the threshold values of | log2FC| > 1.5 and FDR < 0.05. Comparing the two lines within the four intestinal compartments, 122 DEG were common to all of four compartments, and 157 DEG to the three small intestinal tissues (Supplementary Figure 7). To verify the results from mRNA-seq, a total of 18 DEG (six upregulated DEG and six downregulated DEG in each compartment; each verified DEG was used in at least two different compartments) were randomly selected and confirmed with qRT-PCR assay. A high Pearson correlation coefficient (0.87) and R-squared value (0.76) were obtained between qRT-PCR assay and mRNA-seq results, demonstrating the reliability of our DEG identification (Supplementary Figure 7).

**FIGURE 4:**
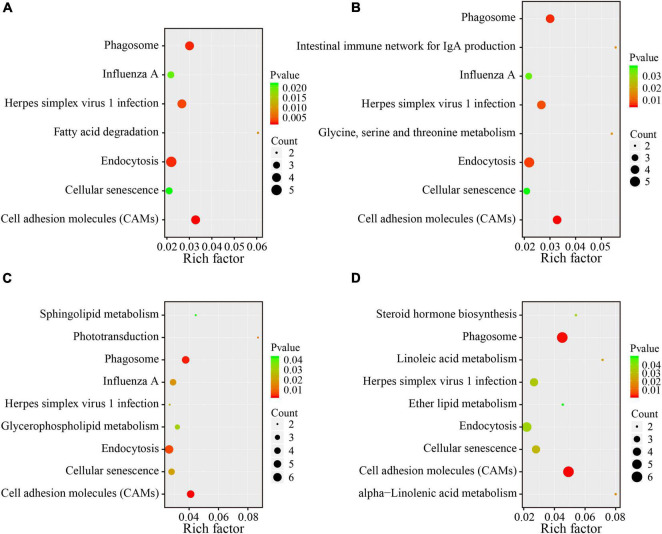
KEGG pathway enrichment analysis of DEG between the two lines in four different intestinal compartments. The *y*-axis shows the name of the pathway, and the *x*-axis shows the Rich factor. The pathways with significant enrichment were shown in the KEGG scatter plot. The dot size represents the number of different genes, and the color indicates the *p*-value. The Rich factor is the proportion of the number of differentially expressed genes and the number of all annotated genes in a given pathway. The greater the Rich factor, the higher the degree of enrichment. **(A)** Duodenum; **(B)** jejunum; **(C)** ileum; **(D)** ceca.

To reveal the underlying functional mechanisms of the differences between the lean and obese broilers, KEGG enrichment analysis was applied to functionally annotate the DEG. Pathway significant enrichment was conducted using pathway profiles in the KEGG database, and hypergeometric tests were performed to examine the pathways that were significantly enriched by the DEG when compared to the background. The significantly enriched pathways were displayed in Figure 4, which illustrates that five pathways were shared in all four compartments. Notably, lipid metabolism-related pathways were significantly changed in some intestinal segments between the two lines (*p* < 0.05), including “fatty acid degradation” in the duodenum; “glycerophospholipid metabolism and sphingolipid metabolism” in the ileum; and “steroid hormone biosynthesis,” “linoleic acid metabolism,” “ether lipid metabolism,” and “alpha-linolenic acid metabolism” in the ceca. The detailed DEG with significant enrichment in lipid metabolism relevant pathways are summarized in Supplementary Table 7. The enrichment analysis demonstrated that the variations of lipid metabolism relevant pathways could be one of the main drivers in regulating the difference of AFD between the two lines.

### Associations of Microbe–Intestine Interactions With Broiler Abdominal Fat Deposition

**FIGURE 5:**
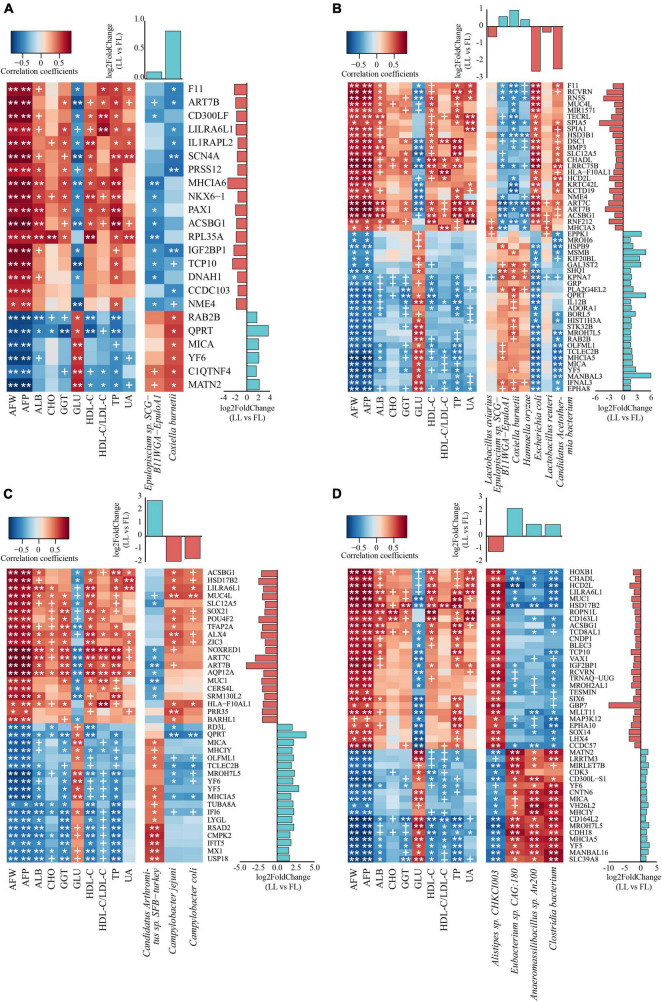
Multi-omics correlation analysis reveals the interactions between microbiome and host. Each heat map contains two parts: the correlations between gene expression and abdominal fat relevant traits (left part of each panel) and correlations between gene expression and microbial relative abundance (right part of each panel). For each tissue compartment, the genes shown had significant correlations and were DEG in the transcriptome analysis. The upper histogram shows the log2FC of microbial relative abundance between the two lines, while the right histogram displays the log2FC of gene expression [lean line (LL) vs. fat line (FL)]. In the histograms, the light blue and orange columns represent genes or microbes significantly enriched in the lean- and fat-line broilers, respectively. The significance *p*-value threshold was set to 0.05 in the small intestine **(A–C)** and 0.01 in the ceca **(D)**. ALB, albumin; CHO, total cholesterol; GGT, γ-glutamyl transpeptidase; GLU, glucose; HDL-C, high-density lipoprotein cholesterol; LDL-C, low-density lipoprotein cholesterol; TP, total protein; UA, uric acid. +*p*-value < 0.1; *p-value < 0.05; **p-value < 0.01. AFW, abdominal fat weight; AFP, abdominal fat percentage. **(A)** Duodenum; **(B)** jejunum; **(C)** ileum; **(D)** ceca.

To further explore the interactions between host and gut microbiome, correlation analysis of abdominal fat traits, intestinal transcriptome, and metagenome species were performed by using a multi-omics correlation analysis. Briefly, the WGCNA R package was used to identify the crucial intestinal genes (Supplementary Figure 2 and Supplementary Table 2) and microbes (Supplementary Figure 3) that significantly correlated with the AFRT within each tissue compartment. Subsequently, based on the AFRT-correlated genes and microbes, correlations between intestinal genes and microbes were calculated (Figure 5). Because of the importance of DEG, the genes in the microbe–gene correlations were required to also have significance as DEG within the tissue compartment; thus, Figure 5 shows the AFRT-correlated DEG in each tissue that were significantly correlated with AFRT-correlated microbial species.

In the duodenum, two microbial species (*Epulopiscium* sp. *SCG-B11WGA-EpuloA1* and *Coxiella burnetii*) that both increased in the lean line were significantly correlated with AFRT (*p* < 0.05, Supplementary Figure 3). When the two microbial species were tested for correlation with the intestinal genes that had significant correlations with AFRT, the expression of 23 duodenal DEG had significant correlations with one or both of the two microbial species relative abundances (*p* < 0.05, Figure 5A). Pathway annotation of these 23 genes showed that they were mainly involved in fatty acid metabolism, transport and catabolism, immunity and infection, and nucleic acid metabolism (Supplementary Table 8). In the jejunum, seven microbial species (*Lactobacillus aviaries*, *Lactobacillus reuteri*, *Epulopiscium* sp. *SCG-B11WGA-EpuloA1*, *C. burnetii*, *Hannaella oryzae*, *E. coli*, and *Candidatus Acetothermia bacterium*) were significantly correlated with AFRT (*p* < 0.05, Supplementary Figure 3). The differential comparison of these seven species between the two lines showed that the abundances of *Epulopiscium* sp. *SCG-B11WGA-EpuloA1*, *C. burnetii*, and *H. oryzae* were higher in the lean line, while the other four species abundances were lower. Within the AFRT-correlated genes, the expression levels of 50 jejunal DEG were subsequently found to have significant correlations with the above seven microbial species (*p* < 0.05, Figure 5B). Notably, *ACSBG1*, *GRP*, *HSD3B1*, and *PLA2G4EL2* genes were involved in lipid metabolic pathways (i.e., “fatty acid biosynthesis,” “fatty acid degradation,” “PPAR signaling pathway,” “adipocytokine signaling pathway,” “steroid hormone biosynthesis,” and “ether lipid metabolism”). In the ileum, three microbial species, *Candidatus Arthromitus* sp. *SFB-turkey*, *Campylobacter jejuni*, and *Campylobacter coli*, were significantly correlated with AFRT (*p* < 0.05, Supplementary Figure 3). Of the three species, the abundances of the two *Campylobacter* species were decreased in the lean line, while the abundance of *Candidatus Arthromitus* sp. *SFB-turkey* was increased. The correlations between these three species and AFRT-correlated genes showed that the expression of 38 ileal DEG had significant correlations with the relative abundance of the three microbial species (*p* < 0.05), where 18 genes were upregulated and the other 20 genes were downregulated (lean line vs. fat line, Figure 5C).

Because of the large number of cecal microbes, only those microbes that were significantly different in the ceca between the two lines were chosen to correlate with AFRT, and four species (*Alistipes* sp. *CHKCI003*, *Eubacterium* sp. *CAG:180*, *Anaeromassilibacillus* sp. *An200*, and *Clostridia bacterium*) were significantly correlated with AFRT (*p* < 0.05, Supplementary Figure 3). In regard to the relative abundance of these four species, *Alistipes* sp. *CHKCI003* was increased in the fat line, while the other three species were decreased. WGCNA identified 44 cecal DEG that significantly correlated with the AFRT and significantly correlated with the above four microbial species (*p* < 0.05, Figure 5D). Of these 44 DEG, 17 genes were upregulated, and the remaining 27 genes were downregulated (lean line vs. fat line). Pathway annotation showed that these correlated genes mainly participated in lipid metabolism, amino acid metabolism, immune system, transport and catabolism, and cell growth and death (Supplementary Table 8).

## Discussion

Recently, studies have characterized the composition and function variation of the gut microbiome in obese individuals, which revealed strong correlations between gut microbiota and obesity (Turnbaugh et al., 2006, 2009; Bouter et al., 2017). However, due to the complexity, high dimensionality, and spatial heterogeneity of the gut microbiome, studies on interactions between gut microbiome and host in obesity are still rare. The application of multi-omics associations provides novel opportunities to uncover the complex host–gut microbiota interactions. To investigate the interactions between gut microbiome and host in obesity, multi-omics associations of gut metagenome, intestinal transcriptome, and abdominal fat relevant phenotypes were performed in a unique NEAUHLF broiler population, which is an excellent obese–lean study model generated by over 20 years of genetic selection for divergent abdominal fat content.

An accurate gut MGC is essential in metagenome sequence analysis and critical for avoiding bias of results. The gut MGC of humans (Qin et al., 2010), mice (Xiao et al., 2015), and pigs (Xiao et al., 2016) have been published in succession. Recently, Huang et al. published the chicken gut MGC by metagenomics sequencing of 495 chickens of all intestinal compartments, which contained 9.04 million non-redundant MG (Huang et al., 2018). A comprehensive comparison of varied gut MGC demonstrated that there is only a relatively small set of shared gut MG in different animal species (Huang et al., 2018). Indeed, gut microbiome depended on many factors like diet, environment and genetics, which demonstrated that varied populations of the same species could harbor varied gut microbiota (Turnbaugh et al., 2009; Gao et al., 2018). Thus, in the current study, an appropriate gut MGC for the lean and fat broilers based on our own metagenome data was constructed and compared to the published chicken MGC. Although lower numbers of non-redundant MG were identified in our MGC (2.44 million vs. 9.04 million), our MGC annotated more specific genera and species for our broilers (Supplementary Figure 8). The smaller number of non-redundant MG in our MGC may be due to the lower sample numbers and single chicken breed in our study. Importantly, the dominant four phyla annotated by our MGC corresponded with those in the published chicken MGC. In addition, the gut MGC construction of our broiler population could also be a supplement for the comprehensive intestinal MGC of chicken, which could benefit deeper exploration in gut microbiome studies.

In the present study, we found that the microbial features of four intestinal compartments as well as their gene functions were distinctly different in lines that had undergone long-term selection for high or low AFD. The comparison of microbial diversity and composition showed differences between the lean- and fat-line broilers, although less difference was detected in the small intestine than in the ceca. Obesity is reported to be accompanied by a decrease in gut microbial diversity and varied microbial structure, mainly caused by gut inflammatory responses (Turnbaugh et al., 2008; De Wit et al., 2012; Hou et al., 2016). Our ceca results were consistent with these findings. A smaller difference in the small intestine might suggest less influence of host genetics on small intestinal microbiome (Wen et al., 2019). Even so, we identified two jejunal species of *E. coli* and *Candidatus Acetothermia bacterium* that were significantly increased in the fat line. *E. coli*, which is often involved in inflammation, has been shown to be enriched in the human gut with non-alcoholic fatty liver disease (Loomba et al., 2017). Obesity is often associated with low-grade inflammation in the intestine (Brestoff and Artis, 2015), which may relate to the increased *E. coli* in fat-line broilers. *Acetothermia* has been viewed as a harmful taxon, associated with diarrhea (Duan et al., 2020). As an opportunistic pathogen, *Acetothermia* may have similar effects as *E. coli* in obesity. We identified six differential bacterial species in the ceca between the lean- and fat-line broilers, four of which (*Alistipes* sp. *CHKCI003*, *R. bacterium CPB6*, *C. bacterium*, and *Anaeromassilibacillus* sp. *An200*) reached significant levels. The short-chain fatty acids (SCFAs) usually fermented by gut microbes mainly include acetate, propionate, and butyrate, which have a diverse array of metabolic effects on host (Bouter et al., 2017). The *Alistipes* sp. has been previously predicted as a microbial marker of obesity and was characterized by an increased distribution in the gut of obese individuals (Louis et al., 2016; Kang et al., 2019). *Alistipes* is an acetic acid producer, which could improve lipid metabolism by its metabolite (Yin et al., 2018). *Ruminococcaceae* members are butyrate producers, which can provide more energy for enterocytes (Wong et al., 2006). Because of butyrate production, *Ruminococcaceae* serve as a healthy bacterium, and its proportion was found to decrease in obese mice (Daniel et al., 2014). However, *R. bacterium CPB6* mainly produces caproate, which had positive relations with BMI (Zhu et al., 2017; Fan et al., 2018). Similarly, *R. bacterium* has also been identified to significantly increase in cecum of high AFD chicken (Xiang et al., 2021). Increased abundances of *Alistipes* sp. *CHKCI003* and *R. bacterium CPB6* in the ceca of the fat-line broilers may be vital factors contributing to AFD with their specific metabolites. *Clostridiales* members have been characterized as defenders against obesity in high fat diet induced mice, which is mediated by their metabolites, including butyrate (Pérez-Matute et al., 2015). *Anaeromassilibacillus*, like *Clostridiales*, is a SCFA producer and protected mice against obesity (Treichel et al., 2019). Here, the lean-line broilers with significantly enriched *C. bacterium* and *Anaeromassilibacillus* sp. *An200* may help to maintain a healthier gut environment and less AFD. Another two cecal species (*Pseudoflavonifractor* sp. *An184* and *Eubacterium* sp. *CAG:180*) showed a trend of difference between the two lines and have been consistently reported as probiotics (Udayappan et al., 2016; Qu et al., 2019). The current study’s results comprehensively compared the relative abundance of microbiota in the four intestinal compartments under the obese chicken model, which has expanded knowledge of gut microbiota variations in obesity.

In addition, the gut metagenome datasets also offered functional microbiome information of the lean- and fat-line broilers, which could supply the extra functions that were not encoded by the host genome (Schmidt et al., 2018). Previously, it reported that the enhanced nucleic acids metabolism were accompanied by increased microbial activities (Morán et al., 2007), while increased microbial carbohydrate metabolism is commonly associated with overproduction and accumulation of TG (Bäckhed et al., 2004). In the study, the MG related to nucleic acids metabolism and carbohydrate metabolism were significantly enriched in the duodenum and jejunum of the fat line, which demonstrated that the microbiome in obese broilers’ duodenum and jejunum might have more metabolism activities and carbohydrate metabolism. Fatty acids are important components of varied lipids, and they are usually increasingly absorbed in the small intestine of obese women (Koffert et al., 2018). Interestingly, MG involved in fatty acid biosynthesis were significantly enriched in the jejunum of fat line, which could add more fatty acid derived from gut microbiome for obese broilers. The amino acids derived from gut microbiota, especially aromatic and branched-chain amino acids, have been thought to be vital modulators in obesity and insulin resistance (Neis et al., 2015; Pedersen et al., 2016). Consistently, the comparison of KEGG pathways and eggNOG ortholog groups in the jejunum showed that MG of amino acid-related metabolism were significantly enriched in the fat-line broilers, which revealed that the specific amino acids produced by jejunal microbiota might contribute to more AFD in obese broilers. However, more significantly enriched microbial functional categories were identified in the ileum and ceca of the lean-line broilers. This may be related to less digestion and absorption in the duodenum and jejunum of the lean-line broilers than fat line, which could leave more substrates for microbiome in the ileum and ceca. Additionally, non-obese individuals harbored more abundant hindgut microbiome with less inflammation than obese ones, which have a higher fermentative capability to maintain balance microenvironment and offer extra energy for the host (Turnbaugh et al., 2009; Ding et al., 2016; Hou et al., 2016; Liu et al., 2016). Similarly, microbiota functions of energy metabolism and carbohydrates metabolism were significantly enriched in lean-line ceca, which could promote anti-inflammatory responses to reduce AFD in the lean broilers. Our functional metagenome profile demonstrated that varied intestinal compartments enriched various microbiome functions in response to divergent AFD in the two lines of broilers.

Previously, many researchers have characterized the variation of intestinal transcriptome in obese animals, especially lipid metabolism-related pathways (Kondo et al., 2006; De Wit et al., 2008; Mao et al., 2013). In the present study, intestinal transcriptome was profiled and compared between the lean and fat lines. Functional enrichments of DEG in tissues of four gut compartments by KEGG analysis showed that seven lipid-related metabolism pathways were significantly varied between the lean- and fat-line broilers. As the intestine is a site for processing exogenous fat (Sklan et al., 1975), functional changes of intestinal lipid metabolism may be among the mechanisms of differences in AFD. Additionally, variations in immune and inflammation-related functions have been identified in obese individuals (Teixeira et al., 2012; Araujo et al., 2017). In the present study, we also found similar changes in pathways mainly involved in immune, infectious disease, and defense responses, and amino acid metabolism between the two lines. Recently, RNA-seq analysis of Chinese meat-type chickens during development demonstrated that genes involved in endocytosis, transcript factors, and cell process-related pathways had strong associations with AFW (Xing et al., 2020). Our study also found some DEG significantly enriched in similar pathways. These transcriptome results reflected that the difference of lipid metabolism, inflammatory, endocytosis, transcript factors, and cell process-related pathways that happened in the intestine may be close to the divergent AFD in the two lines of broilers.

Multi-omics association analyses were performed using AFRT, intestinal gene expression, and microbial species abundance, to determine the correlations existing between AFRT-correlated DEG and microbial species, which could influence broiler AFD. In the duodenum, *Epulopiscium* sp. *SCG-B11WGA-EpuloA1* was significantly negatively correlated with genes involved in transport and catabolism (*MHCIA6*), lipid metabolism (*ACSBG1*), and nucleotide metabolism (*NME4*). *Epulopiscium* sp. was detected commonly in fish intestine and could decrease digestive enzyme activity by regulating intestinal pH (Pollak and Montgomery, 1994). Increased abundance of *Epulopiscium* sp. *SCG-B11WGA-EpuloA1* may downregulate catabolism and lipid metabolism-related genes by inhibiting lipase activity in the lean line duodenum. Another species, *C. burnetii*, was mainly positively correlated with infectious disease-related genes like *RAB2B*, *MICA*, *C1QTNF4* (Day et al., 2018), and *YF6* (Thoraval et al., 2003). Although *C. burnetii* has been viewed as pathogenic, its avirulent strain does not cause symptoms in the host but may instead promote immune system development (Long et al., 2019). Infectious disease-related gene expression increased with increased *C. burnetii* abundance in the lean-line duodenum, which may be a mechanism to avoid endotoxemia-mediated obesity by maintaining integrity of the intestinal mucosal barrier. However, further studies and evidence are needed to prove the strong correlations between the specific strain of *C. burnetii* and obesity.

In the jejunum, four microbial species enriched in the obese line were correlated with AFRT-correlated genes; *E. coli* and *Candidatus Acetothermia bacterium* have more correlated genes, while *L. aviaries* and *L. reuteri* were both correlated with one gene (*MHCIA3*). As mentioned above, *E. coli* and *Candidatus Acetothermia bacterium* are opportunistic pathogens and were significantly increased in obese jejunum. These bacteria can injure the intestinal mucosa barrier and induce lipopolysaccharide (LPS)-mediated endotoxemia in obesity (Schwiertz et al., 2010a; Loomba et al., 2017; Lee et al., 2018; Duan et al., 2020). Consistently, more immune relevant genes (*MICA*, *MHCIA5*, and *RAB2B*) were negatively correlated with these two species. *E. coli* has also been reported to induce an increase in fatty acid absorption by competing for monosaccharides and disaccharides with epithelial cells (Tazi et al., 2018). In our study, lipid metabolism-related genes like *ACSBG1* and *HSD3B1* were significantly increased in the fat line and have significantly positive correlations with *E. coli*. For the two *Lactobacillus* species increased in the fat line, their functions are still undetermined because inconsistent variation was identified in the obese mouse (Jiao et al., 2018). Few correlations were identified between *Lactobacillus* spp. and intestinal genes, which suggests that *Lactobacillus* spp. do not play a dominant regulatory role in our broilers. Besides, two microbes, *Epulopiscium* sp. *SCG-B11WGA-EpuloA1 and C. burnetii*, enriched in the jejunum and duodenum of lean line had analogously related genes. Thus, we speculated that there are similar interactions between the lean line enriched microbes and DEG in both the duodenum and jejunum.

In the ileum, *C. jejuni* and *C. coli* were enriched in the fat line, positively correlated with two lipid metabolism-related genes (*ACSBG1* and *HSD17B2*), and strongly negatively correlated with the *QPRT* gene. *Campylobacter*, as the cause of human campylobacteriosis, is widespread in poultry in which it is a commensal (Williams et al., 2016). *Campylobacter* can also cause inflammation in the intestine, disturbing the gut microenvironment and injuring gut barrier functions (Knudsen et al., 2006). Here, correlations between lipid metabolic-related genes and *Campylobacter* sp. in the ileum showed similar impacts of opportunistic pathogen on lipid metabolism as observed in the duodenum and jejunum. Immune-related genes, like quinolinate phosphoribosyl transferase (*QPRT*), protect against oxidative stress (Kibi et al., 2019). Increased abundance of *Campylobacter* sp. may aggravate the inflammatory response by downregulating the *QPRT* gene. Only one lean-line enriched species was identified in the ileum, *Candidatus Arthromitus* sp. *SFB-turkey*, which had negative correlations with transport relevant genes [i.e., *ART7C*, *ART7B* (Lesma et al., 1998), and *AQP12A*] and positive correlations with immune-related genes [*YF5*, *MICA* (Yuan et al., 2019), *RSAD2*, and *MX1*]. *Candidatus Arthromitus* sp. was reported to be important to the maturation of innate and adaptive immune functions in the murine gut (Bolotin et al., 2014). The increased abundance of *Candidatus Arthromitus* sp. may reflect a well-functioning ileum in the lean line, with potentially greater nutrition absorption and immune maturation.

Chicken ceca function as a fermentative and immune-related organ (Huang et al., 2018). For fat-line enriched species in the ceca, *Alistipes* sp. *CHKCI003* was significantly positively correlated with *ACSBG1*, *HSD17B2* (involved in lipid metabolism) and *CNDP1* (amino acid metabolism). As previously mentioned, *Alistipes* sp. is an acetic acid-producing bacteria and associated with enhanced lipid metabolism in obese mice (Louis et al., 2016; Yin et al., 2018; Kang et al., 2019). The fat-line ceca had increased abundance of *Alistipes* sp. *CHKCI003* correlated with upregulated genes in lipid metabolism. Expression of the other 15 DEG (mainly involved in the immune system) was negatively correlated with *Alistipes* sp. *CHKCI003* abundance, which might be associated with inflammation in fat-line ceca. In addition, three lean line enriched species (*Eubacterium* sp. *CAG:180*, *Anaeromassilibacillus* sp. *An200*, and *C. bacterium*) were positively correlated with immune relevant genes. The three species are SCFA producers, which benefit healthier enteric performance (Qu et al., 2019; Treichel et al., 2019). Their metabolites, SCFAs, have been proven to maintain a lower pH value and more energy, which could inhibit pathogen colonization (Venegas et al., 2019). Thus, we speculate that immune-related DEG interacting with SCFAs-producing bacteria drives anti-inflammatory responses to prevent obesity. However, many DEG with no functional annotation were also identified to have correlations with AFRT-correlated microbial species, which suggests that comprehensive interactions exist between gut microbiota and host genes. More research is needed to fully characterize the interactions between gut microbiota and host.

## Conclusion

**FIGURE 6:**
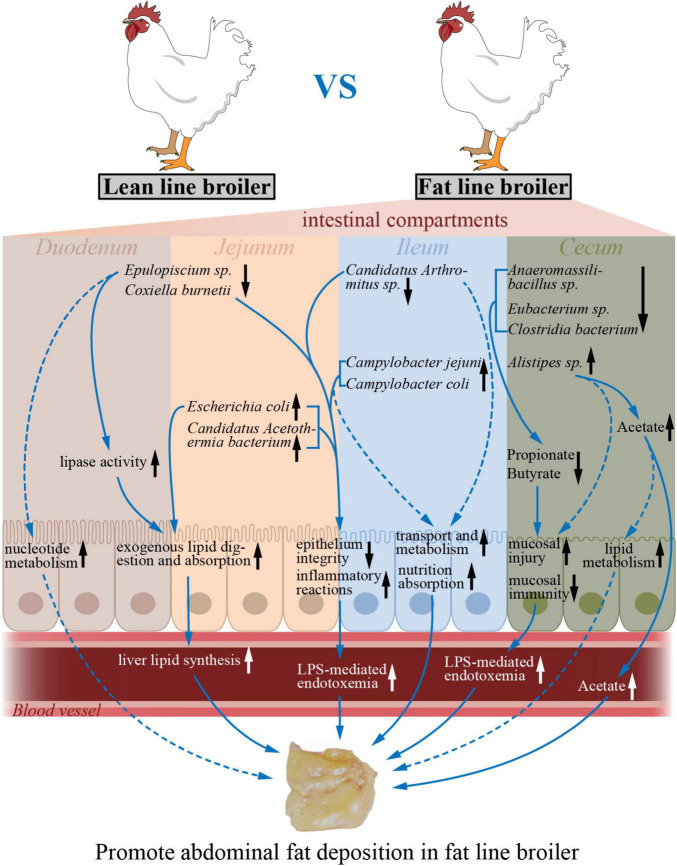
The potential model of how varied microbes impacted by host genetic selection regulate intestinal functions to promote abdominal fat deposition. The up and down arrows (black) indicate increased and decreased intestinal functions or microbial abundances in the fat-line broilers compared to the lean-line broilers. The dotted arrows (blue) designate those changes that are predicted by intestinal transcriptome–microbiome interactions. The solid arrows (blue) represent the regulatory effects of the microbes and functions.

In conclusion, a microbial gene reference catalog of the NEAUHLF population was constructed, which provides a supplement for the published chicken gut microbiome. With the divergently selection for abdominal fat, the differences of microbial composition in cecum between the two lines of broilers were larger than that in the small intestine. When compared to lean-line broilers, the microbial richness and diversity were significantly decreased in the ceca of fat-line broilers, but there were no differences in the small intestine. The intestinal functions reflected by transcriptome were significantly varied as the more AFD in fat line. Multi-omics associations demonstrated that specialized gut microbiota, like *E. coli*, *Candidatus Acetothermia bacterium*, and *Alistipes* sp. *CHKCI003*, which varied by intestinal compartment and by chicken line, had significant correlations with corresponding intestinal genes involved in lipid metabolism, immune system, transport, and catabolism-related pathways, which suggests that microbiome–host interactions may contribute to AFD (Figure 6). Our study, therefore, expands knowledge of the relationship between obesity and gut microbiome. Future association studies with more omics datasets (like metabolome, proteome, and metatranscriptome) will help us to better uncover in detail the complex interaction mechanisms between gut microbiome and intestinal genes.

## Data Availability Statement

The datasets presented in this study can be found in online repositories. The names of the repository/repositories and accession number(s) can be found below: NCBI BioProject - PRJNA779845, Gene Expression Omnibus - GSE179459.

## Ethics Statement

The animal study was reviewed and approved by Laboratory Animal Management Committee of Northeast Agricultural University. Written informed consent was obtained from the owners for the participation of their animals in this study.

## Author Contributions

HL contributed to the conceptualization. HL and HY contributed to the project administration and funding acquisition. HL, SL, and HY contributed to the supervision. YJ and HL contributed to the writing original draft. YJ, YY, PW, HL, HY and NW contributed to the methodology. YJ, YY, and PW contributed to the formal analysis and software. YJ contributed to the investigation and visualization. YJ and HL contributed to the validation experiments. HL, MM, SL, and YJ contributed to the review and editing. YJ, FM, QZ, WN, KZ, YW, LL, and HY contributed to the sampling and resources. YL and PL contributed to the animal administration. YY, NW, and RG contributed to the advices supplement. All authors approved the final manuscript.

## Conflict of Interest

YY, PW, and RG were employed by the company Novogene Bioinformatics Technology Co. The remaining authors declare that the research was conducted in the absence of any commercial or financial relationships that could be construed as a potential conflict of interest.

## Publisher’s Note

All claims expressed in this article are solely those of the authors and do not necessarily represent those of their affiliated organizations, or those of the publisher, the editors and the reviewers. Any product that may be evaluated in this article, or claim that may be made by its manufacturer, is not guaranteed or endorsed by the publisher.

## Footnotes

1
https://github.com/cjfields/readfq

2
http://topaz.gatech.edu/GeneMark/

3
https://github.com/bbuchfink/diamond/

4
https://www.ncbi.nlm.nih.gov/

5
https://www.moma.dk/files/r.NormOldStab5.txt

## Supplementary Material

Supplementary Figure 1 Phenotype correlations of serum biochemical indices. Pearson correlations were performed between abdominal fat traits and serum biochemical indices. The upper numbers were the correlation coefficients, while the lower numbers were corresponding *P* values. Serum biochemical indices and numbers with bold fonts were significantly correlated with abdominal fat traits (*P* < 0.05), and were clustered into abdominal fat relevant traits (AFRT) for the following association analysis. AFW, abdominal fat weight; AFP, abdominal fat percentage; TG, triglycerides; CHO, total cholesterol; HDL-C, high-density lipoprotein cholesterol; LDL-C, low-density lipoprotein cholesterol; TBA, total bile acid; TP, total protein; ALB, albumin; GLU, glucose; AST, aspartate transaminase; ALT, alanine transaminase; CREA, creatinine; GGT, γ-glutamyl transpeptidase; UA, uric acid.

Supplementary Figure 2 Correlation analysis between abdominal fat relevant traits (AFRT) and intestinal gene modules. WGCNA methods were performed to identify the vital genes that have impacts on abdominal fat deposition. Genes were first clustered into modules based on their expression variations. Correlations between AFRT and modules were subsequently determined, and modules with correlation *P* values less than 0.05 were selected. Genes within the significant modules with correlation *P* values less than 0.05 were identified as AFRT-correlated genes. AFW, abdominal fat weight; AFP, abdominal fat percentage; ALB, albumin; CHO, total cholesterol; GGT, γ-glutamyl transpeptidase; GLU, glucose; HDL-C, high-density lipoprotein cholesterol; LDL-C, low-density lipoprotein cholesterol; TP, total protein; UA, uric acid.

Supplementary Figure 3 Correlation analysis between abdominal fat relevant traits (AFRT) and microbial relative abundance. Spearman correlation was performed and the microbial species with correlation *P* values less than 0.1 were selected as vital AFRT-correlated species. Due to the much larger number of species in the ceca, only species with differential abundance between the two lines were correlated with the AFRT, and *P* value threshold was set to 0.01. AFW, abdominal fat weight; AFP, abdominal fat percentage; ALB, albumin; CHO, total cholesterol; GGT, γ-glutamyl transpeptidase; GLU, glucose; HDL-C, high-density lipoprotein cholesterol; LDL-C, low-density lipoprotein cholesterol; TP, total protein; UA, uric acid.

Supplementary Figure 4 The rarefaction analysis of the identified genes from the total set of 80 samples **(E)** and the subgroups of duodenum **(A)**, jejunum **(B)**, ileum **(C)**, and ceca **(D)** compartments. A total of 2.44 million non-redundant genes were identified, and the rarefaction curve including all samples approached saturation by 20 samples. The gene number for a specific number of samples was calculated after random samplings repeated 100 times with replacement, and the median was plotted.

Supplementary Figure 5 The difference of genera between the lean- and fat-line broilers. Microbial genera identified in all samples of at least one group with relative abundance over 0.1% were used to perform the differential comparison between the two lines. Metastats software was performed and genera with FDR < 0.1 were shown in the figure. No differential genera between the two broiler lines were identified in duodenum and ileum. The green and orange columns stand for the lean and fat line, respectively. *FDR < 0.05; ^+^0.05 ≤ FDR < 0.1.

Supplementary Figure 6 The ratio of Firmicutes to Bacteroidetes between the two lines. A declined ratio of these two phyla was observed in all four compartments of obese broilers, which expanded the previous knowledge on variation of intestinal microbiome in obese individuals. LL, lean line; FL, fat line.

Supplementary Figure 7 Identification and comparison of DEG between two broiler lines within four intestinal compartments. **(A)** Volcano plots of gene expression changes between the two lines for duodenum, jejunum, ileum and ceca compartments (from left to right, respectively). The threshold values were | log2FC| > 1.5 and FDR < 0.05. The red dots and green dots represent up- and down-regulated genes, respectively. The black dots signify genes with no statistically significant difference. **(B)** Venn diagram of DEG overlapping across the four intestinal compartments. **(C)** Linear regression fitted for log2FC of selected genes determined via qRT-PCR assay and mRNA-Seq technology. R-square and Pearson correlation coefficient are labeled as “*R*^2^” and “*r.*” Different contrasts of the four intestinal compartments were marked with different shapes. Log2FC in qRT-PCR equals −ΔΔCt for each comparison. Average Ct value for each group was the means of samples in that group. The housekeeping gene of *TBP* was used for normalization of Ct values.

Supplementary Figure 8 The annotated comparison of microbiome reference gene catalog to the published one. The green area (located in the left site) represents the published catalog by Huang et al. (2018), while the red area (located in the right site) represents our catalog. **(A)** Venn diagram of annotated phyla present in and shared by two reference gene catalogs. **(B)** Venn diagram of annotated genera present in and shared by two reference gene catalogs. **(C)** Venn diagram of annotated species present in and shared by two reference gene catalogs. **(D)** Venn diagram of KEGG orthologous groups (KOs) present in and shared by two reference gene catalogs.

Supplementary Table 1 Primers of validation qPCR experiment.

Supplementary Table 2 Intestinal genes that significantly correlated with abdominal fat relevant traits.

Supplementary Table 3 The detailed data yield of whole metagenome seqeuncing.

Supplementary Table 4 The basic assembly statistics of metagenomic shotgun sequencing data.

Supplementary Table 5 The information of taxonomical annotation in the constructed gut microbial gene catalog.

Supplementary Table 6 The basic statistics of mRNA-Seq data.

Supplementary Table 7 The DEG significantly enriched in lipid metabolism relevant pathways.

Supplementary Table 8 KEGG pathways annotations of the DEG that significantly correlated with microbial specices.

## Abbreviations

AFRT: abdominal fat relevant traits
DEG: differentially expressed gene
AFD: abdominal fat deposition
NEAUHLF: Northeast Agricultural University broiler lines divergently selected for abdominal fat content
AFW: abdominal fat weight
AFP: abdominal fat percentage
BW: body weight
TG: triglycerides
CHO: total cholesterol
HDL-C: high-density lipoprotein cholesterol
LDL-C: low-density lipoprotein cholesterol
TBA: total bile acid
TP: total protein
ALB: albumin
GLU: glucose
AST: aspartate transaminase
ALT: alanine transaminase
CREA: creatinine
GGT: γ-glutamyl transpeptidase
UA: uric acid
MGC: microbial gene catalog.
