# Supplementary figures and images for: Multi-Omics Association Reveals the Effects of Intestinal Microbiome–Host Interactions on Fat Deposition in Broilers
Source: Front Microbiol. 2022 Feb 17;12:815538. doi: 10.3389/fmicb.2021.815538 (PMC8892104; doi:10.3389/fmicb.2021.815538)

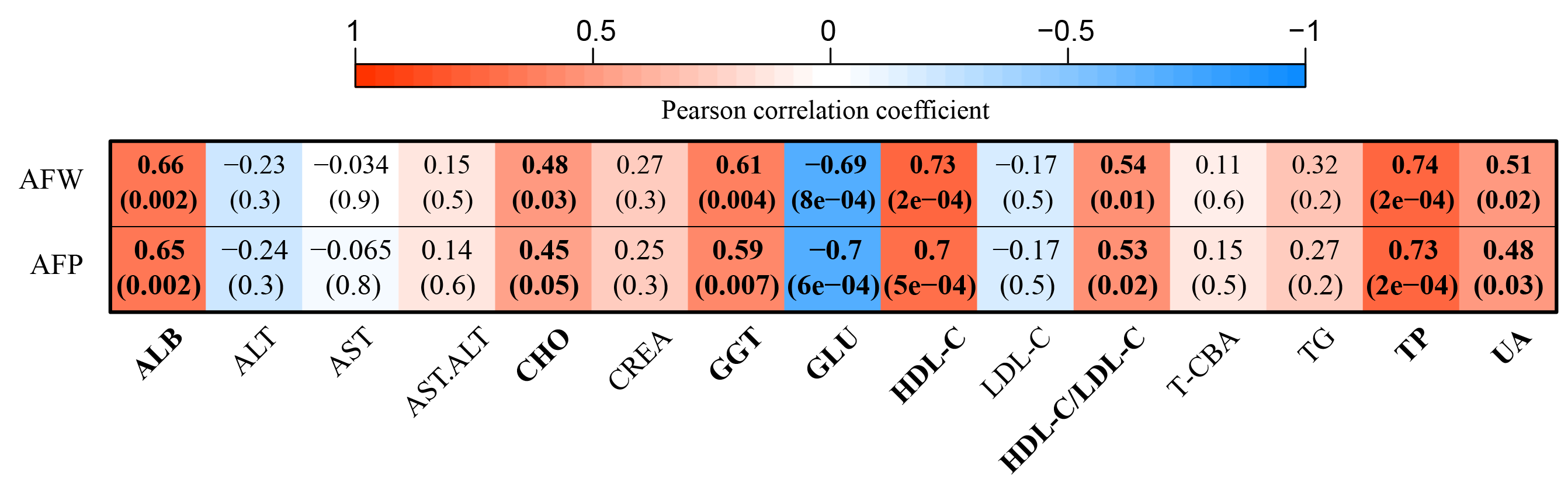

Supplement: Supplementary Figure 1 — Phenotype correlations of serum biochemical indices. Pearson correlations were performed between abdominal fat traits and serum biochemical indices. The upper numbers were the correlation coefficients, while the lower numbers were corresponding P values. Serum biochemical indices and numbers with bold fonts were significantly correlated with abdominal fat traits (P < 0.05), and were clustered into abdominal fat relevant traits (AFRT) for the following association analysis. AFW, abdominal fat weight; AFP, abdominal fat percentage; TG, triglycerides; CHO, total cholesterol; HDL-C, high-density lipoprotein cholesterol; LDL-C, low-density lipoprotein cholesterol; TBA, total bile acid; TP, total protein; ALB, albumin; GLU, glucose; AST, aspartate transaminase; ALT, alanine transaminase; CREA, creatinine; GGT, γ-glutamyl transpeptidase; UA, uric acid. [file Data_Sheet_1.ZIP › Supplementary Materials/Supplementary Figure S1.tif]

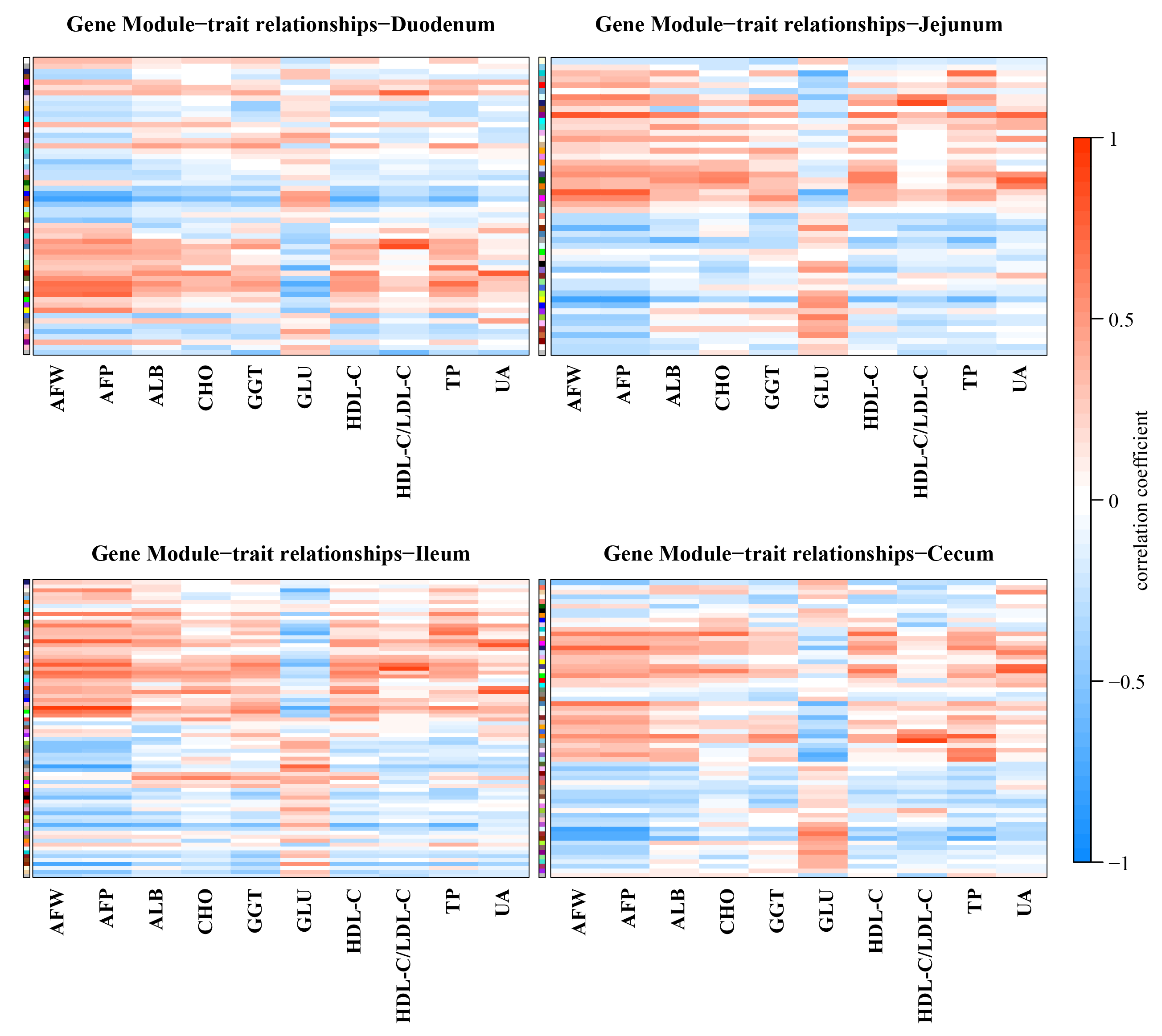

Supplement: Supplementary Figure 1 — Phenotype correlations of serum biochemical indices. Pearson correlations were performed between abdominal fat traits and serum biochemical indices. The upper numbers were the correlation coefficients, while the lower numbers were corresponding P values. Serum biochemical indices and numbers with bold fonts were significantly correlated with abdominal fat traits (P < 0.05), and were clustered into abdominal fat relevant traits (AFRT) for the following association analysis. AFW, abdominal fat weight; AFP, abdominal fat percentage; TG, triglycerides; CHO, total cholesterol; HDL-C, high-density lipoprotein cholesterol; LDL-C, low-density lipoprotein cholesterol; TBA, total bile acid; TP, total protein; ALB, albumin; GLU, glucose; AST, aspartate transaminase; ALT, alanine transaminase; CREA, creatinine; GGT, γ-glutamyl transpeptidase; UA, uric acid. [file Data_Sheet_1.ZIP › Supplementary Materials/Supplementary Figure S2.tif]

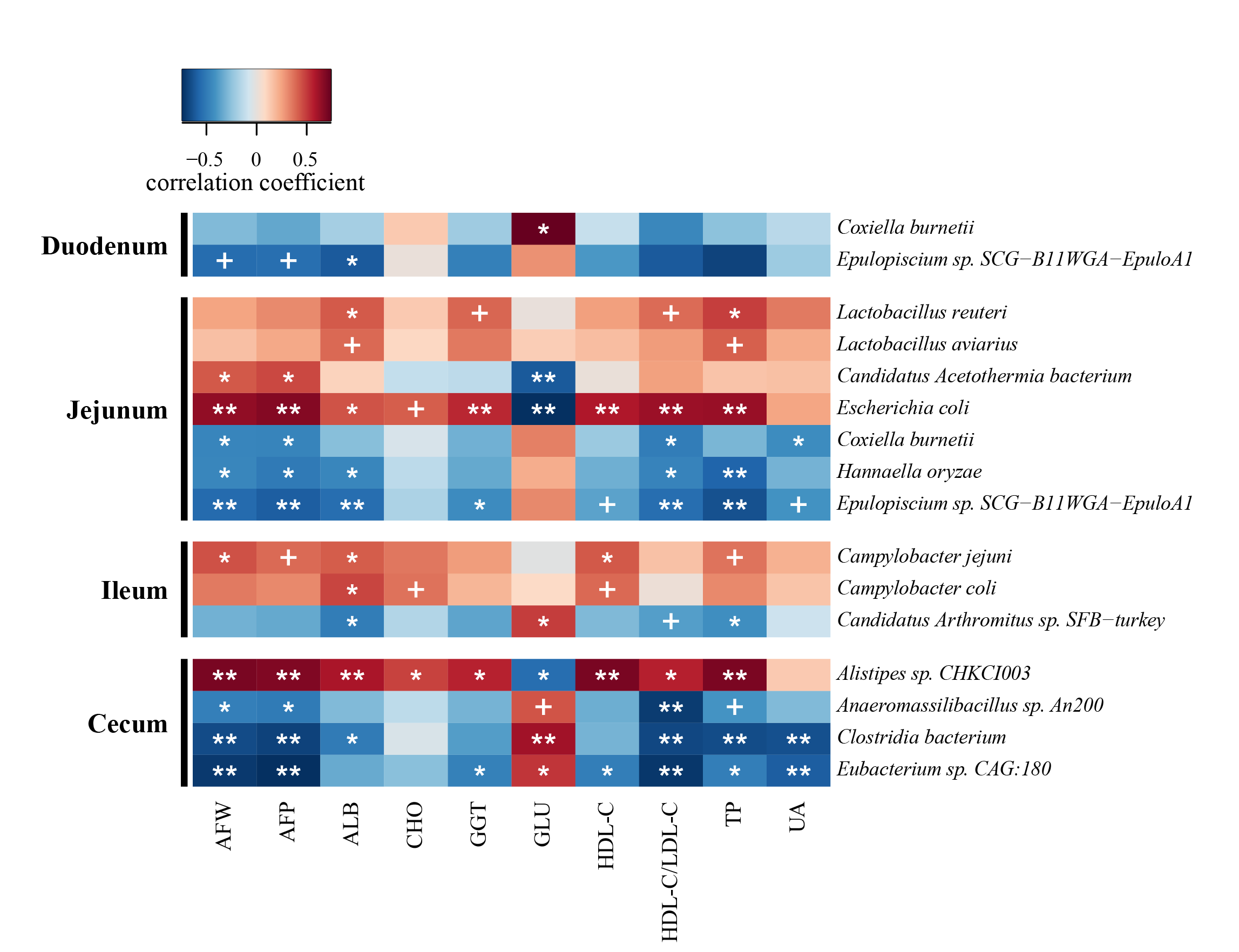

Supplement: Supplementary Figure 1 — Phenotype correlations of serum biochemical indices. Pearson correlations were performed between abdominal fat traits and serum biochemical indices. The upper numbers were the correlation coefficients, while the lower numbers were corresponding P values. Serum biochemical indices and numbers with bold fonts were significantly correlated with abdominal fat traits (P < 0.05), and were clustered into abdominal fat relevant traits (AFRT) for the following association analysis. AFW, abdominal fat weight; AFP, abdominal fat percentage; TG, triglycerides; CHO, total cholesterol; HDL-C, high-density lipoprotein cholesterol; LDL-C, low-density lipoprotein cholesterol; TBA, total bile acid; TP, total protein; ALB, albumin; GLU, glucose; AST, aspartate transaminase; ALT, alanine transaminase; CREA, creatinine; GGT, γ-glutamyl transpeptidase; UA, uric acid. [file Data_Sheet_1.ZIP › Supplementary Materials/Supplementary Figure S3.tif]

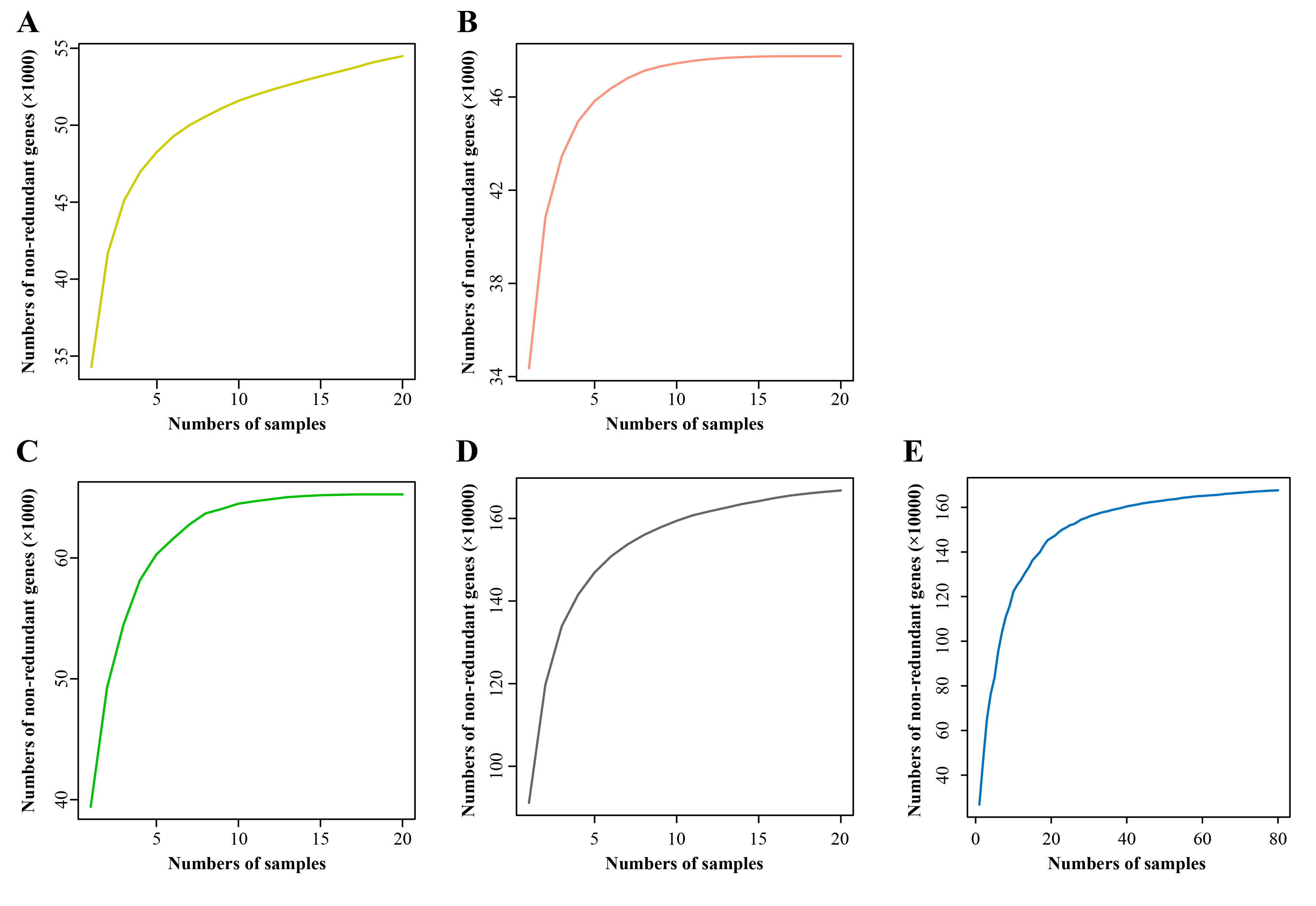

Supplement: Supplementary Figure 1 — Phenotype correlations of serum biochemical indices. Pearson correlations were performed between abdominal fat traits and serum biochemical indices. The upper numbers were the correlation coefficients, while the lower numbers were corresponding P values. Serum biochemical indices and numbers with bold fonts were significantly correlated with abdominal fat traits (P < 0.05), and were clustered into abdominal fat relevant traits (AFRT) for the following association analysis. AFW, abdominal fat weight; AFP, abdominal fat percentage; TG, triglycerides; CHO, total cholesterol; HDL-C, high-density lipoprotein cholesterol; LDL-C, low-density lipoprotein cholesterol; TBA, total bile acid; TP, total protein; ALB, albumin; GLU, glucose; AST, aspartate transaminase; ALT, alanine transaminase; CREA, creatinine; GGT, γ-glutamyl transpeptidase; UA, uric acid. [file Data_Sheet_1.ZIP › Supplementary Materials/Supplementary Figure S4.tif]

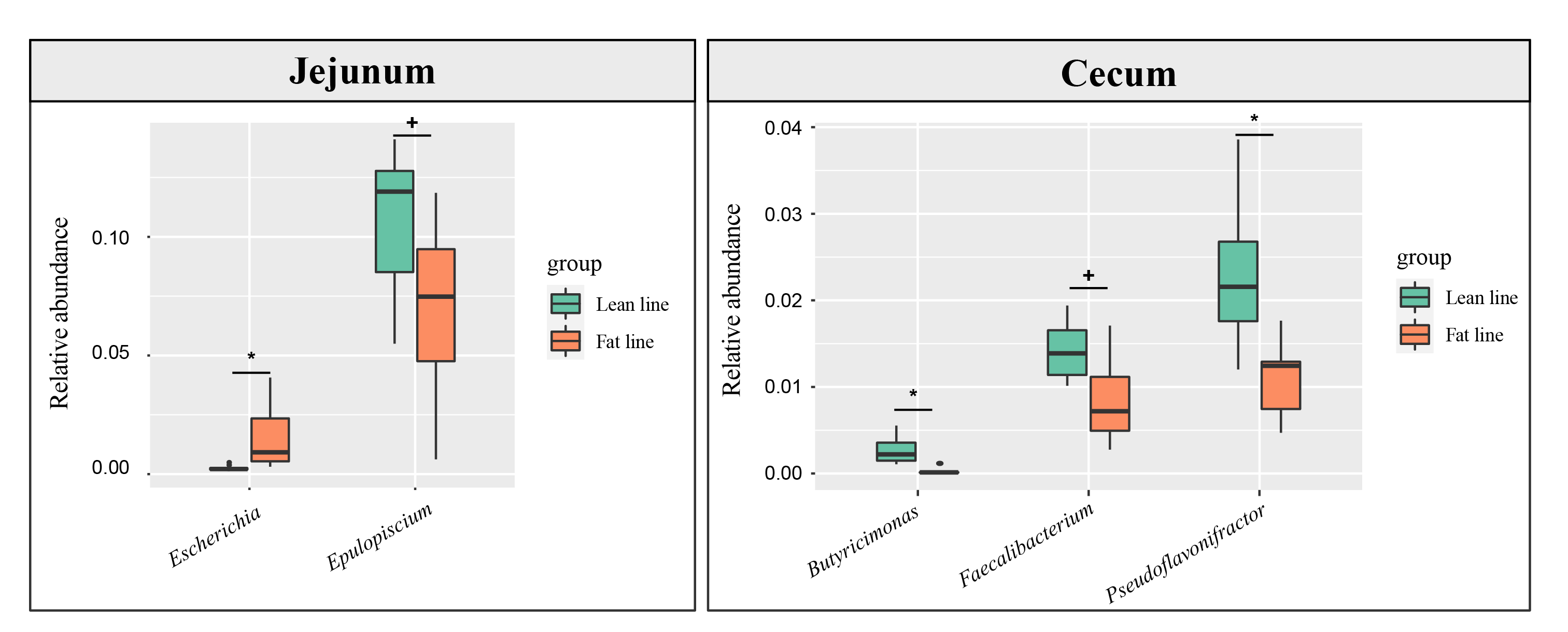

Supplement: Supplementary Figure 1 — Phenotype correlations of serum biochemical indices. Pearson correlations were performed between abdominal fat traits and serum biochemical indices. The upper numbers were the correlation coefficients, while the lower numbers were corresponding P values. Serum biochemical indices and numbers with bold fonts were significantly correlated with abdominal fat traits (P < 0.05), and were clustered into abdominal fat relevant traits (AFRT) for the following association analysis. AFW, abdominal fat weight; AFP, abdominal fat percentage; TG, triglycerides; CHO, total cholesterol; HDL-C, high-density lipoprotein cholesterol; LDL-C, low-density lipoprotein cholesterol; TBA, total bile acid; TP, total protein; ALB, albumin; GLU, glucose; AST, aspartate transaminase; ALT, alanine transaminase; CREA, creatinine; GGT, γ-glutamyl transpeptidase; UA, uric acid. [file Data_Sheet_1.ZIP › Supplementary Materials/Supplementary Figure S5.tif]

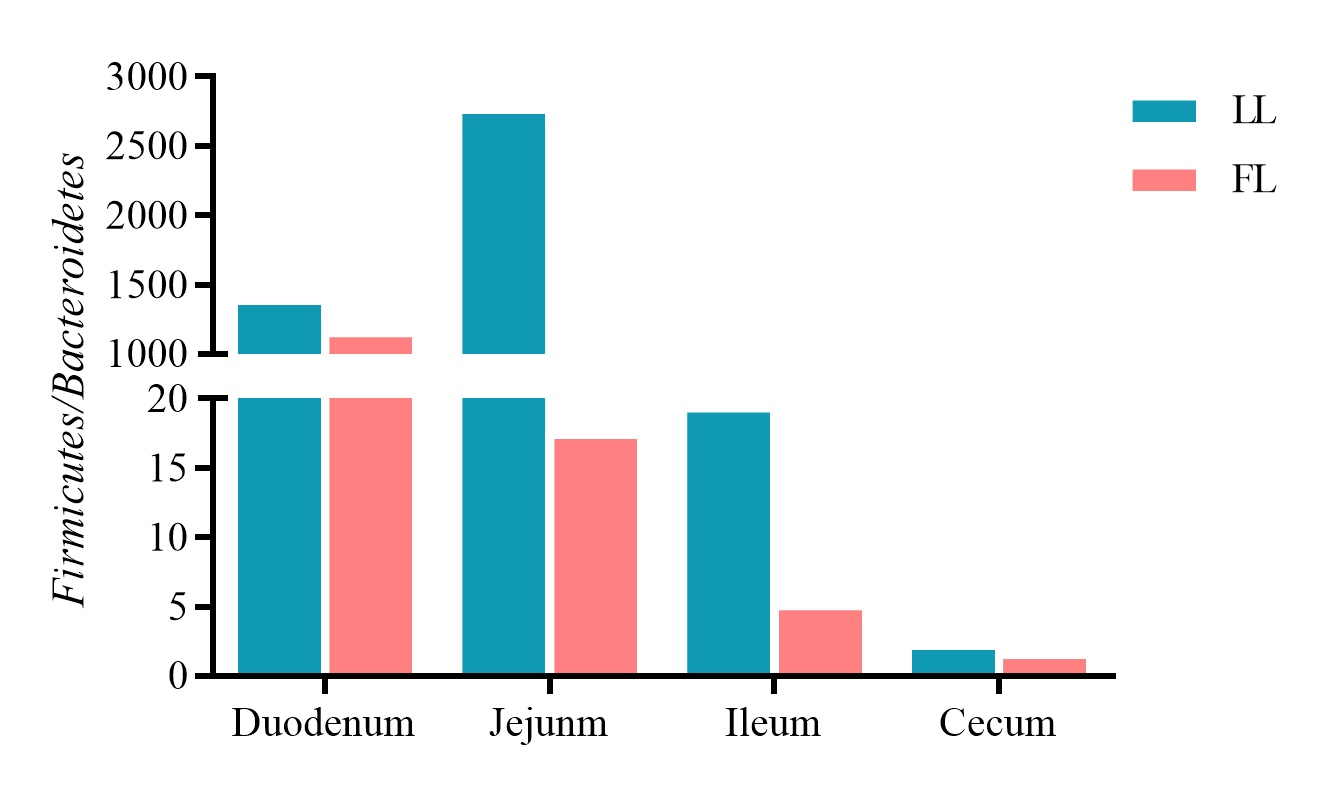

Supplement: Supplementary Figure 1 — Phenotype correlations of serum biochemical indices. Pearson correlations were performed between abdominal fat traits and serum biochemical indices. The upper numbers were the correlation coefficients, while the lower numbers were corresponding P values. Serum biochemical indices and numbers with bold fonts were significantly correlated with abdominal fat traits (P < 0.05), and were clustered into abdominal fat relevant traits (AFRT) for the following association analysis. AFW, abdominal fat weight; AFP, abdominal fat percentage; TG, triglycerides; CHO, total cholesterol; HDL-C, high-density lipoprotein cholesterol; LDL-C, low-density lipoprotein cholesterol; TBA, total bile acid; TP, total protein; ALB, albumin; GLU, glucose; AST, aspartate transaminase; ALT, alanine transaminase; CREA, creatinine; GGT, γ-glutamyl transpeptidase; UA, uric acid. [file Data_Sheet_1.ZIP › Supplementary Materials/Supplementary Figure S6.tif]

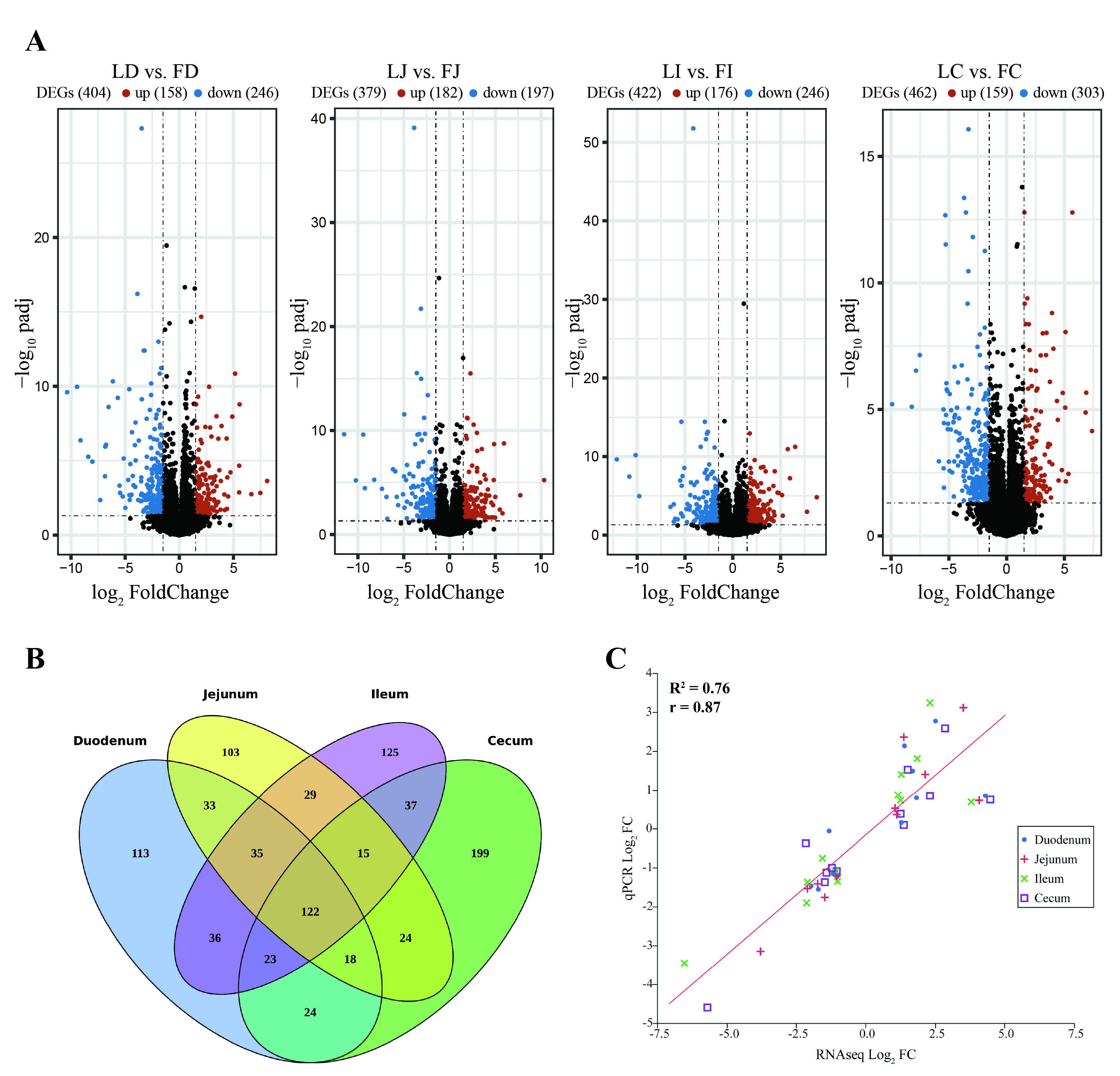

Supplement: Supplementary Figure 1 — Phenotype correlations of serum biochemical indices. Pearson correlations were performed between abdominal fat traits and serum biochemical indices. The upper numbers were the correlation coefficients, while the lower numbers were corresponding P values. Serum biochemical indices and numbers with bold fonts were significantly correlated with abdominal fat traits (P < 0.05), and were clustered into abdominal fat relevant traits (AFRT) for the following association analysis. AFW, abdominal fat weight; AFP, abdominal fat percentage; TG, triglycerides; CHO, total cholesterol; HDL-C, high-density lipoprotein cholesterol; LDL-C, low-density lipoprotein cholesterol; TBA, total bile acid; TP, total protein; ALB, albumin; GLU, glucose; AST, aspartate transaminase; ALT, alanine transaminase; CREA, creatinine; GGT, γ-glutamyl transpeptidase; UA, uric acid. [file Data_Sheet_1.ZIP › Supplementary Materials/Supplementary Figure S7.tif]

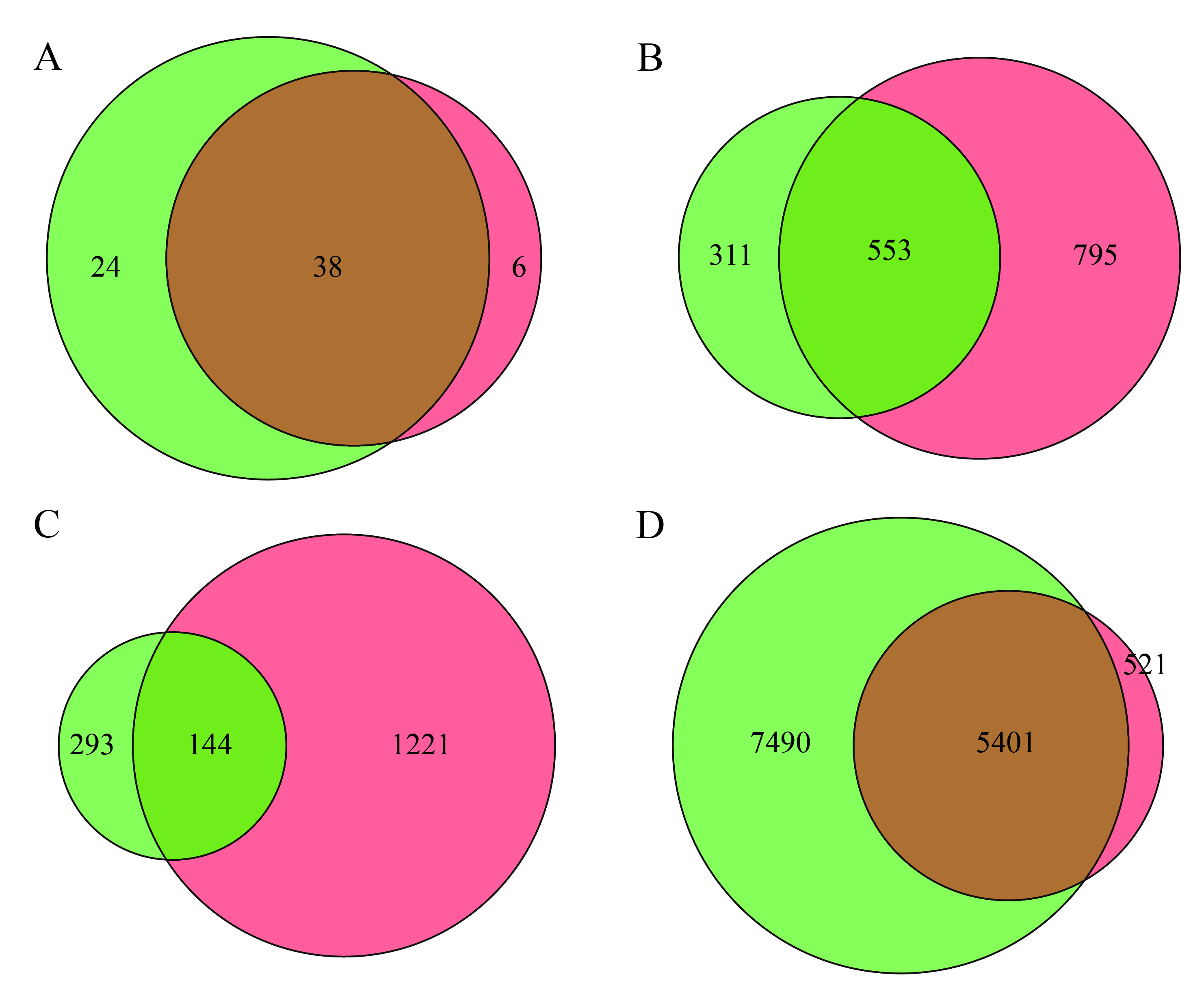

Supplement: Supplementary Figure 1 — Phenotype correlations of serum biochemical indices. Pearson correlations were performed between abdominal fat traits and serum biochemical indices. The upper numbers were the correlation coefficients, while the lower numbers were corresponding P values. Serum biochemical indices and numbers with bold fonts were significantly correlated with abdominal fat traits (P < 0.05), and were clustered into abdominal fat relevant traits (AFRT) for the following association analysis. AFW, abdominal fat weight; AFP, abdominal fat percentage; TG, triglycerides; CHO, total cholesterol; HDL-C, high-density lipoprotein cholesterol; LDL-C, low-density lipoprotein cholesterol; TBA, total bile acid; TP, total protein; ALB, albumin; GLU, glucose; AST, aspartate transaminase; ALT, alanine transaminase; CREA, creatinine; GGT, γ-glutamyl transpeptidase; UA, uric acid. [file Data_Sheet_1.ZIP › Supplementary Materials/Supplementary Figure S8.tif]
